# Characterization of Potent Fusion Inhibitors of Influenza Virus

**DOI:** 10.1371/journal.pone.0122536

**Published:** 2015-03-24

**Authors:** Michael Rowse, Shihong Qiu, Jun Tsao, Tongmei Xian, Sarah Khawaja, Yohei Yamauchi, Zhen Yang, Guoxin Wang, Ming Luo

**Affiliations:** 1 Department of Microbiology, University of Alabama at Birmingham, 1025 18^th^ Street South, Birmingham, Alabama, 35294, United States of America; 2 Laboratory of Structural Biology, School of Chemical Biology and Biotechnology, Peking University Shenzhen Graduate School, Shenzhen, 518055, China; 3 Institute of Biochemistry, ETH Zurich HPM E8.2, Otto-Stern-Weg 3, 8093 Zurich, Switzerland; 4 Laboratory of Chemical Genomics, School of Chemical Biology and Biotechnology, Peking University Shenzhen Graduate School, Shenzhen, 518055, China; 5 Department of Chemistry, College of Arts and Sciences, Georgia State University, Atlanta, Georgia, 30302, United States of America; German Primate Center, GERMANY

## Abstract

New inhibitors of influenza viruses are needed to combat the potential emergence of novel human influenza viruses. We have identified a class of small molecules that inhibit replication of influenza virus at picomolar concentrations in plaque reduction assays. The compound also inhibits replication of vesicular stomatitis virus. Time of addition and dilution experiments with influenza virus indicated that an early time point of infection was blocked and that inhibitor **136** tightly bound to virions. Using fluorescently labeled influenza virus, inhibition of viral fusion to cellular membranes by blocked lipid mixing was established as the mechanism of action for this class of inhibitors. Stabilization of the neutral pH form of hemagglutinin (HA) was ruled out by trypsin digestion studies *in vitro* and with conformation specific HA antibodies within cells. Direct visualization of **136** treated influenza virions at pH 7.5 or acidified to pH 5.0 showed that virions remain intact and that glycoproteins become disorganized as expected when HA undergoes a conformational change. This suggests that exposure of the fusion peptide at low pH is not inhibited but lipid mixing is inhibited, a different mechanism than previously reported fusion inhibitors. We hypothesize that this new class of inhibitors intercalate into the virus envelope altering the structure of the viral envelope required for fusion to cellular membranes.

## Introduction

Influenza virus is an enveloped virus belonging to the *Orthomyxoviridae* family. Waterfowls are the natural reservoir for most influenza A subtypes. Avian influenza viruses bind with high affinity to α2,3 linked sialic acid containing receptors and with low affinity to α2,6 linked receptors, the converse applies for human influenza viruses [1]. Species such as pigs that contain both α2,3 and α2,6 linked receptors allow coinfection with both human and avian influenza viruses [2]. Genome reassortment of coinfecting influenza viruses may result in a new influenza virus strain containing different subtypes of HA (hemagglutinin) or NA (neuraminidase) previously unseen in humans. Humans may not have preexisting immunity to a new strain of influenza virus, so pandemics can result from genome reassortment [3]. Human cases of H5N1 have occurred sporadically since 1997 and in 2013 human cases of H7N9 have been reported [4]. Both H5N1 and H7N9 are highly pathogenic in humans and are currently circulating in avian reservoirs [4]. The potential of H5N1 or H7N9 viruses to jump to humans emphasize the need for broad spectrum influenza inhibitors since vaccine development would require months. Considering the possibility of increased resistance to neuraminidase inhibitors [5, 6], and the threat of avian viruses to gain transmissibility among humans, new influenza inhibitors are urgently needed.

Fusion inhibitors have been successfully used in the treatment of HIV [7]. For instance, enfuvirtide is a peptide derived from gp41 that blocks refolding of gp41, effectively arresting fusion of HIV to the cell membrane [8–10]. A peptide based inhibitor with a cholesterol moiety attached has successfully targeted influenza virus fusion *in vitro* [11]. LJ001, a small molecule able to inhibit fusion of many pseudotyped enveloped viruses, proves that small molecules can block the fusion pathway of viruses [12]. If the influenza virus fusion pathway could be targeted effectively by small molecule inhibitors, these inhibitors could become an important new class of inhibitors for controlling influenza virus.

A potent inhibitor of influenza virus, (Z)-3-(bicyclo[2.2.1]heptan-2-yl)-5-((5-(4’-chlorophenyl)-3-(3-(piperazin-1-yl)pentyl)furan-2-yl)-methylene)-2-thioxothiazolidin-4-one, (named compound **136**, S1 File) was developed recently [13], but the mechanism of inhibition by **136** was not clearly defined. Here we report that **136** interferes with the fusion process of influenza virus, likely by disrupting the structure of the viral envelope necessary for fusion to cellular membranes.

## Materials and Methods

### Cells and viruses

MDCK-2 cells were cultured in EMEM supplemented with 5% FBS and penicillin/streptomycin. The cells were maintained in a humidified incubator at 37°C, with 5% CO_2._ All influenza viruses were grown in MDCK-2 cells. Influenza virus strain X-31 (H3N2) was amplified by infecting confluent MDCK-2 cells at an MOI of 0.001. After two days post-infection the supernatant from the cell culture was collected and subject to centrifugation at 2000 RCF to remove cell debris and the virus in the supernatant was pelleted at 60,000 RCF for 1 hour. The virus pellet was resuspended in 10 mM HEPES, 100 mM NaCl, pH 7.5 and further purified on a 20–50% sucrose gradient by centrifugation for 1.75 hours at 60,000 RCF. The fractions containing X-31 virus were collected and diluted with 10 mM HEPES, 100 mM NaCl pH 7.5 buffer. The virus was pelleted by centrifugation at 60,000 RCF for 1 hour. The virus pellet was resuspended at 2 mg/mL in 10 mM HEPES, 100 mM NaCl, pH 7.5 and stored at -80°C. X-31 virus was quantitated using the Bio-Rad protein assay and BSA as a standard. A549 ATCC cells were cultured as previously described [14]. Vesicular stomatitis virus (VSV) Indiana serotype was amplified by infecting confluent HeLa cells at an MOI of 0.001. After 1 day post-infection, the supernatant from the cell culture was collected and subject to centrifugation at 2000 RCF to remove cell debris.

### Antibodies

Anti-HA polyclonal antibodies have been described previously [14].

### MTT assay

MDCK-2 cells were seeded at 4 to 5x10^4^ cells/well on 96-well cell culture plate in 100 μL medium containing 10% fetal bovine serum (FBS) and 1% antibiotic solution. The cells were incubated overnight in a humidified 5% CO_2_ incubator at 37°C. Untreated cells were used as a positive control. A set of wells treated with 1% Triton X-100 was used as 100% toxicity (negative control). The inhibitor solution was added to washed cells at a final volume of 100 μL per well in triplicate for each condition. The cells were incubated for one day in a humidified 5% CO_2_ incubator at 37°C. 15 μL of MTT substrate was added to each well (Promega) and incubated at 37°C for 1–4 hours. The reaction was stopped by adding 100 μL stop solution, following by incubation for 5 hours at room temperature to ensure that the formazan crystal was dissolved. The absorbance at 570 nm was measured by a 96-well plate reader, using a reference wavelength of 720 nm. The EC_50_ value was determined by locating the X-axis value corresponding to one-half the maximum absorbance value.

### Time of addition assay

Confluent MDCK-2 cells in 6 well plates were infected with 1000 pfu of X-31 virus and left for 1 hour at 4°C to synchronize infection. The inoculum was removed and 2 mL of EMEM was added to each well. The samples were then placed at 37°C in a humidified incubator. At the indicated time points, 20 nM of **136** or **211** was added to the wells drop wise and placed back into the incubator. For the -1 hour sample the inoculum was incubated for 1 hour prior to infecting cells. For the 0 hour sample, the inoculum was mixed with inhibitor and immediately added to the cells prior to cold room incubation. After 15 hours the supernatant was removed, serially diluted, and the virus concentration was tittered by plaque assay. The data shown is representative of 3 independent experiments. Plaque assays were performed in duplicate with error bars ± the standard deviation.

### Plaque reduction assay

100 pfu of influenza virus in 100 μL of EMEM was incubated with DMSO, or inhibitors at various concentrations for 1 hour. All samples contained 1% DMSO. Monolayers of MDCK-2 cells in 6 well plates were infected with 100 μL of virus samples and incubated for 1 hour at 37°C. The inoculum was removed from the cells and EMEM supplemented with 0.8% agar and 2 μg/mL TPCK-trypsin was added to each well. After 36 hours the cells were fixed with 4% formaldehyde, the agar plugs removed, and the cells stained with 0.1% crystal violet. For VSV, 100 pfu of virus in 100 μL of DMEM was incubated with DMSO, or inhibitors at various concentrations for 1 hour. All samples contained 1% DMSO. Monolayers of HeLa cells in 6 well plates were infected with 100 μL of virus samples and incubated for 1 hour at 37°C. The inoculum was removed from the cells and DMEM supplemented with 0.8% agar was added to each well. After 24 hours the cells were fixed with 4% formaldehyde, the agar plugs removed, and the cells stained with 0.1% crystal violet. The data shown is representative of 3 independent experiments. Plaque assays were performed in triplicate with error bars ± the standard deviation. Curve fitting, EC_50_ calculation, and 95% confidence intervals were calculated with GraphPad Prism 5 using the log[inhibitor] vs. response equation.

### Multicycle inhibition assay

Monolayers of MDCK-2 cells were infected with X-31 virus. To allow binding and internalization of the virus to occur without exposure to **136**, at 1 hour post-infection **136** (or DMSO as a control) was added to the media at a final concentration of 5 μM, 1μM, or 200 nM. The infected cells were incubated for 24 hours at 37°C and at 12 hours and 24 hours post-infection aliquots were taken for plaque assay. The data shown is representative of 2 independent experiments. Data points are the average of 2 replicates ± SD.

### Preparation of fluorescently labeled virus

X-31 virus was labeled with DiD by directly adding a 5 μL aliquot of DiD Vybrant solution to 500 μL of 2 mg/mL virus sample. Labeling was performed for 2 hours at 37°C with constant shaking. Unincorporated dye was removed by centrifugation at 60,000 x g for 30 minutes and the pellet was resuspended in 10 mM HEPES, 100 mM NaCl, pH 7.5. Virus labeling by DiOC18 was described previously [14].

### Dilution of inhibitor-bound virus assay

1200 pfu of X-31 virus in 50 μL EMEM was incubated for 1 hour with 0.5 μL of 50 nM **136** in DMSO or DMSO at room temperature. The sample was then diluted with EMEM to 1 mL and incubated another hour. The remaining virus titer was determined by plaque assay as described above except that 250 μL of sample was used to inoculate. As a control, 1200 pfu in 1 mL was added to the same quantity of **136** and plaque assayed. The data shown is representative of 3 independent experiments. Each data point is the average of 3 replicates ± the standard deviation.

### X-31 virus binding assay

A549 cells were detached and counted, and incubated with X-31 for 1 hour at 4°C. After washing with cold medium, the cells were fixed with 4% formaldehyde at RT. After washing, the cells were incubated in PBS containing 0.1% saponin, 1% bovine serum albumin, and HA mouse monoclonal antibody HA1 (1:3000) (Banerjee et al., [14]) for 1 hour at room temperature. Cells were incubated with secondary anti mouse IgG-AF647 (Invitrogen) (1:2000) for 30 minutes. 10,000 cells were analyzed using FACS Canto II (BD Biosciences). Similar results were obtained when binding was performed on non-detached A549 cells.

### X-31 virus acidification, and lipid mixing assay

X-31 influenza virus was preincubated for 30 minutes with 2.5 μM **136** or **211**. For the acidification assay, A549 cells were bound with X-31 for 1 hour at 4°C in duplicate. Cells were warmed to 37°C to allow endocytosis of the virus. After 1 hour the cells were washed with phosphate buffered saline (PBS), harvested by trypsinization, and fixed with 4% formaldehyde. After washing, the cells were incubated in PBS containing 0.1% saponin, 1% bovine serum albumin, and HA post-acid conformation specific mouse monoclonal antibody A1 (1:1000) for 1 hour at room temperature. Cells were incubated with secondary anti mouse IgG-AF488 (Invitrogen) (1:2000) for 30 minutes. 10,000 cells were analyzed using FACS Canto II (BD Biosciences). Bafilomycin A1 (BafA) was used at 50 nM as a negative control. The experiment was repeated three times. For the lipid mixing assay, X-31 virus was labeled with R18/SP-DiOC18 [14]. A549 cells were infected for 1 hour as above. For the acid-bypass assay, trypsinized A549 cells were bound with labeled X-31 virus for 45 min on ice, and subsequently incubated for 2 min at 37°C in either pH 6.8 or pH 5.0 medium, and fixed. For FACS analysis the cells were harvested by trypsinization and 5,000 cells were analyzed. For microscopy analysis, cells were grown on coverslips, infected, fixed in 4% formaldehyde, and examined using a Zeiss LSM 510 confocal fluorescence microscope. BafA was used as a negative control. BafA blocks endosome acidification, blocking the low pH-induced conformational change of HA, and lipid mixing of R18/SP-DiOC18 labeled influenza virus in late endosomes.

### Trypsin digestion

A 50 μL aliquot of X-31 virus at 0.08 mg/mL was incubated with DMSO, **211**, or **136** for 1 hour at room temperature. Trypsin (1mg/mL final concentration) was added to the samples. Acidified samples were acidified to pH 5.0 for 5 minutes by addition of 50 mM citrate pH 3.0 100 mM NaCl, reneutralized with 100 μM tris pH 10.0 100 mM NaCl, and left at 37°C for 15 minutes to allow digestion to occur. For samples that were not acidified, an equivalent volume of 10 mM HEPES pH 7.5 100 mM NaCl was added to the samples. Trypsin digestion was stopped by addition of 2 mM 4-(2-Aminoethyl)benzenesulfonyl fluoride hydrochloride (AEBSF) for 15 minutes. Samples were mixed with nonreducing loading buffer and loaded onto a 10% polyacrylamide gel for SDS-PAGE. The gel was fixed and stained with the Pierce Silver Stain Kit and imaged with a ccd based gel imager (Syngene G:box).

### Negative stain electron microscopy

The **136** treated X-31 virus samples were prepared as above without addition of trypsin. 7 μL of sample was applied to a glow discharged carbon coated grid for 30 seconds, blotted with filter paper, stained with 7 μL of 1% phosphotungstic acid pH 7.5 for 20 seconds, and blotted again. Samples were imaged with a FEI Tecnai 12 transmission electron microscope.

## Results

**Fig 1:**
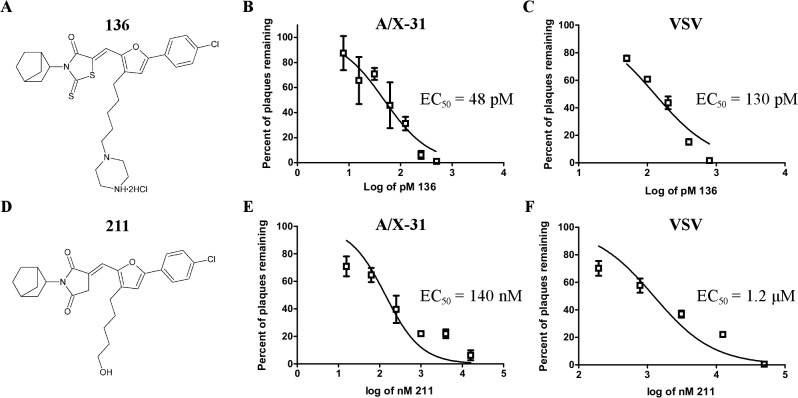
Structure and EC_50_ values of inhibitors. The structures of compounds **136** (A) and **211** (D). X-31 virus plaque reduction assays were performed using monolayers of MDCK-2 cells. For X-31 virus, the EC_50_ of **136** and **211** was calculated to be 48 pM and 140 nM, respectively (B and E). VSV plaque reduction assays were performed using monolayers of HeLa cells. For VSV, the EC_50_ of **136** and **211** was calculated to be 130 pM and 1.2 μM, respectively (C and F). Representative data are shown from 3 independent experiments. Data points are the average of 3 replicates ± SD.

**Fig 2:**
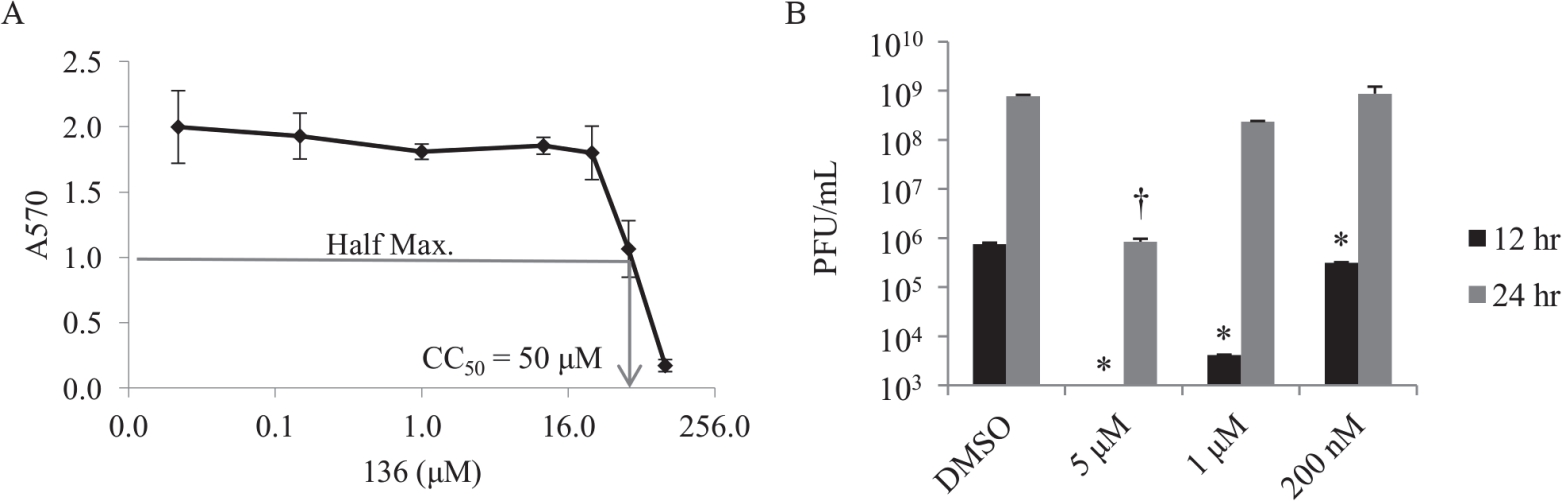
The CC_50_ of 136 is 50 μM and multicycle X-31 virus replication is inhibited. (A) Monolayers of MDCK-2 cells were used to determine the cellular toxicity of **136** by MTT assay. The half maximal response corresponding to the CC_50_ concentration of **136** was determined to be 50 μM. Data shown is the average of 3 replicates ± SD. (B) Monolayers of MDCK-2 cells were used to test if **136** inhibits multicycle replication of X-31 virus. Untreated X-31 virus was added to cells and allowed to enter host cells for 1 hour. Then **136** at a final concentration of 5 μM, 1μM, or 200 nM were added to the cells. Aliquots were taken at 12 and 24 hours post-infection and plaque assayed. After 12 hours of X-31 virus infection, 5 μM, 1μM, and 200 nM **136** containing samples were significantly inhibited as compared to the DMSO control (* p<0.001, one-way ANOVA and Tukey post-hoc test). After 24 hours of X-31 virus infection, 5 μM **136** is significantly inhibited as compared to the DMSO control († p<0.05. one-way ANOVA and Tukey post-hoc test). The data shown is representative of 2 independent experiments. Data points are the average of 2 replicates ± SD.

Compound P25H2 was previously found to inhibit cell infection of multiple influenza virus strains with high potency [15]. Derivatives of P25H2 were synthesized and tested for anti-influenza activities in plaque reduction assays [13]. Fig. 1A shows the structure of a particularly potent derivative, **136**. A less potent derivative (compound **211**) with a similar structure is used for a control because it inhibits virus only at a much higher concentration than **136** (Fig. 1D). Using X-31 virus the EC_50_ values of **136** and **211** were calculated by plaque reduction assays and the results are shown in Fig. 1B and E, respectively. **136** has an EC_50_ value of 48 picomolar whereas **211** has an EC_50_ value of 140 nanomolar, a difference of approximately 2900 fold. Plaque reduction assays with vesicular stomatitis virus (VSV) were also performed with **136** and **211** (Fig. 1C and F). **136** and **211** inhibited VSV with an EC_50_ of 130 pM and 1.2 μM, respectively. An approximately three fold greater concentration of **136** is required for inhibition of VSV as compared to X-31 virus. Table 1 summarizes the EC_50_ of **136** against many other influenza virus strains and includes the 95% confidence interval for all virus strains tested. S1 Fig. shows the plaque reduction assay results for the additional virus strains tested. Table 2 summarizes the EC_50_ values of **211** and includes the 95% confidence interval for the virus strains tested. Additionally, the pH of virus preparations were unaltered by **136** (S1 Table) and the cellular toxicity (CC_50_) of **136** was determined to be 50 μM by a MTT assay (Fig. 2A). The selectivity index of **136** is calculated to be 1x10^6^. Because of the high potency and low cellular toxicity of **136**, it was selected for further characterization.

**Table 1:** 136 Inhibition summary.

Virus	EC_50_ (pM)	95% Confidence interval
A/Udorn/72	60.0	54.0–66.3
A/PR/8/34	27.5	25.1–30.1
A/Aichi/68	209.9	153.6–286.8
A/Victoria/3/75	126.8	98.9–162.5
A/NWS/G70C	45.3	38.7–53.0
A/X-31	48.3	35.5–65.7
B/Lee/40	64.8	53.3–78.8
VSV	127.5	102.2–159.1

**Table 2:** 211 Inhibition summary.

Virus	EC_50_ (nM)	95% Confidence interval
A/X-31	137	81–233
VSV	1240	833–1840

To assess whether multicycle influenza virus infection was inhibited by **136**, infected cells were cultured in growth media supplemented with either DMSO, 5 μM **136**, 1 μM **136**, or 200 nM **136** (Fig. 2B). After 12 or 24 hours of virus growth, aliquots of the media supernatant were plaque assayed. At the 12 hour time point, the 5 μM, 1 μM, and 200 nM **136** samples all significantly reduced the number of plaques as compared with the DMSO control sample (one-way ANOVA and Tukey post hoc test, p<0.001). After 24 hours, the 5 μM **136** sample significantly reduced the number of plaques as compared to the DMSO control sample (one-way ANOVA and Tukey post hoc test p<0.05). **136** inhibits single cycle and multicycle replication of influenza virus.

**Fig 3:**
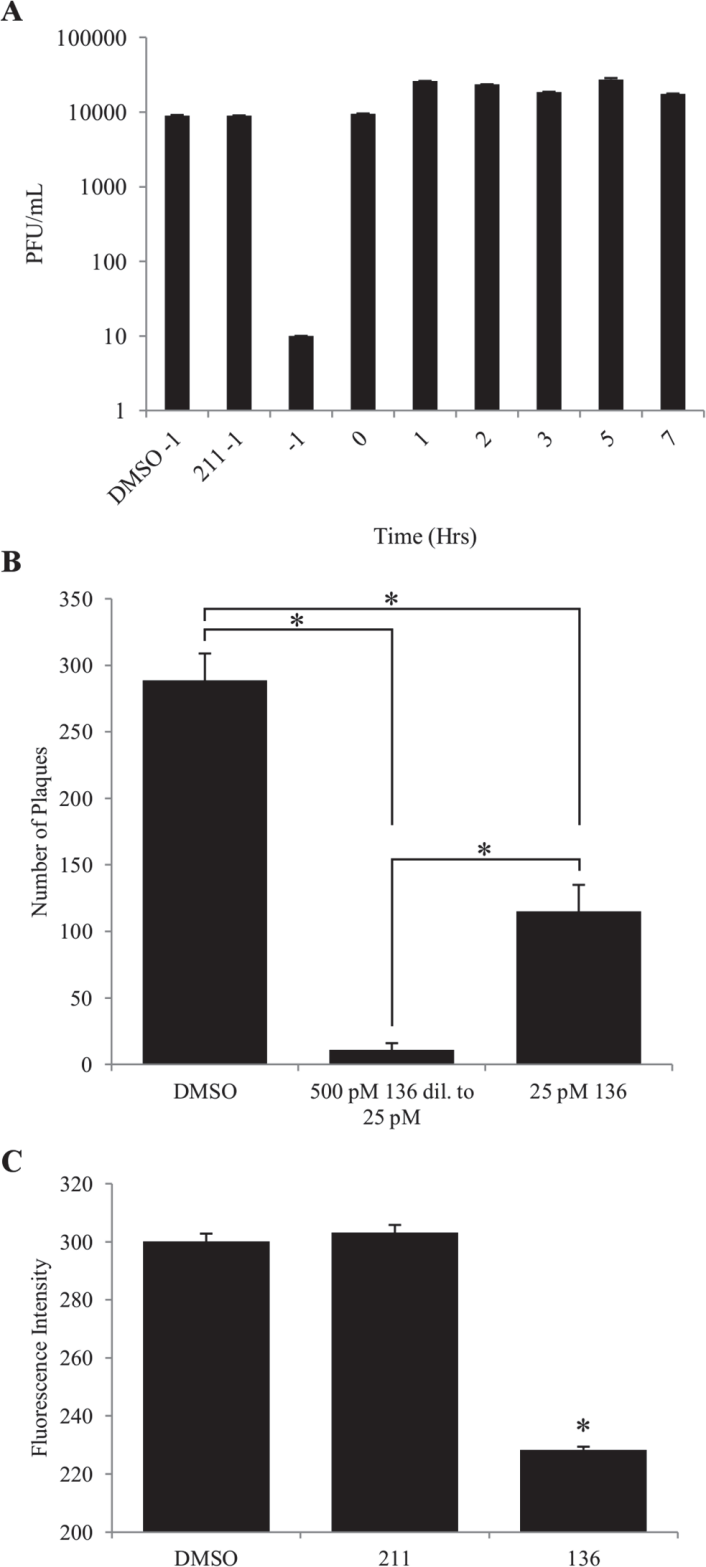
Tight binding to virions. (A) MDCK-2 cells were infected with X-31 virus and treated with **136** at various time points. For-1 hour time points the incoculum was preincubated with **136** then added to cells. After 15 hours the virus titer was calculated by plaque assay. **136** treated X-31 at the-1 hour time point resulted in the greatest inhibition, causing a 3 log reduction in pfu/mL (-1). Controls preincubated with DMSO or **211** do not show inhibition (DMSO-1, and **211**–1). At all other time points when **136** was added (0, 1, 2, 3, 5, 7 hrs) no significant inhibition was observed. (B) **136** is tightly bound to X-31 virus as demonstrated by insensitivity to dilution. As a negative control DMSO was added to virus and diluted 20 fold so that 300 pfu would remain (DMSO). The EC_95_ concentration (500 pM) of **136** was added to virus and diluted 20 fold (500 pM **136** dil to 25 pM). As a positive control virus was first diluted 20 fold and then 25 pM of **136** was added. If binding of **136** was loose, both samples with **136** should cause inhibition to the same extent since **136** would diffuse into the larger volume (* p<0.001, one-way ANOVA and Tukey post-hoc test). (C) X-31 virus was labeled with the fluorescent lipophilic dye DiD. DMSO, **211**, or **136** were added to the labeled X-31 samples and the quenching of DiD was monitored using a spectrofluorometer at λ_EX_ = 644 nm/λ_EM_ = 665 nm. The fluorescence signal was the same for DMSO and **211** treated samples but the fluorescence signal was significantly quenched by **136** (* p<0.001, one-way ANOVA and Tukey post-hoc test). The data shown is representative of 2 independent experiments. Data points are the average of 3 replicates ± SD.

To determine which step of the virus replication cycle **136** most effectively inhibits, time of addition experiments were performed (Fig. 3A). For time of -1 hour the compound was preincubated with virus for 1 hour prior to infection. All other time points indicate hours post-infection that the compound was added. The -1 hour time point for **136** had a 3 log reduction in virus titer. When **136** was added at any other time point there was no inhibition as compared to samples treated with **211** or DMSO. Inhibition of the virus at the-1 hour time point indicates that **136** may bind directly to virions and inhibit a step during virus entry into the host cell.

To confirm that **136** indeed binds to the virion, the EC_95_ concentration of **136** was added to a virus sample and then diluted by 20 fold (Fig. 3B). As a positive control for exchangeable binding, another virus sample was first diluted by 20 fold and then treated with 25 pM of **136**. As a negative control DMSO was added and the sample was diluted by 20 fold. If **136** was to exchangeably bind to the virion, it should diffuse into the larger volume when diluted and inhibit the same amount of virus as the positive control. Fig. 3B shows that even 1 hour after dilution **136** still inhibits 95% of the virus, which is consistent with its tight binding to the virus. Fluorescent labeling of X-31 virus was quantitated in the presence of **136** and **211**, as well as DMSO as a control (Fig. 3C). The fluorescence of lipophilic labeling agent DiD was further quenched below the control background by **136**, suggesting that **136** binds in close proximity to the membrane bound DiD.

**Fig 4:**
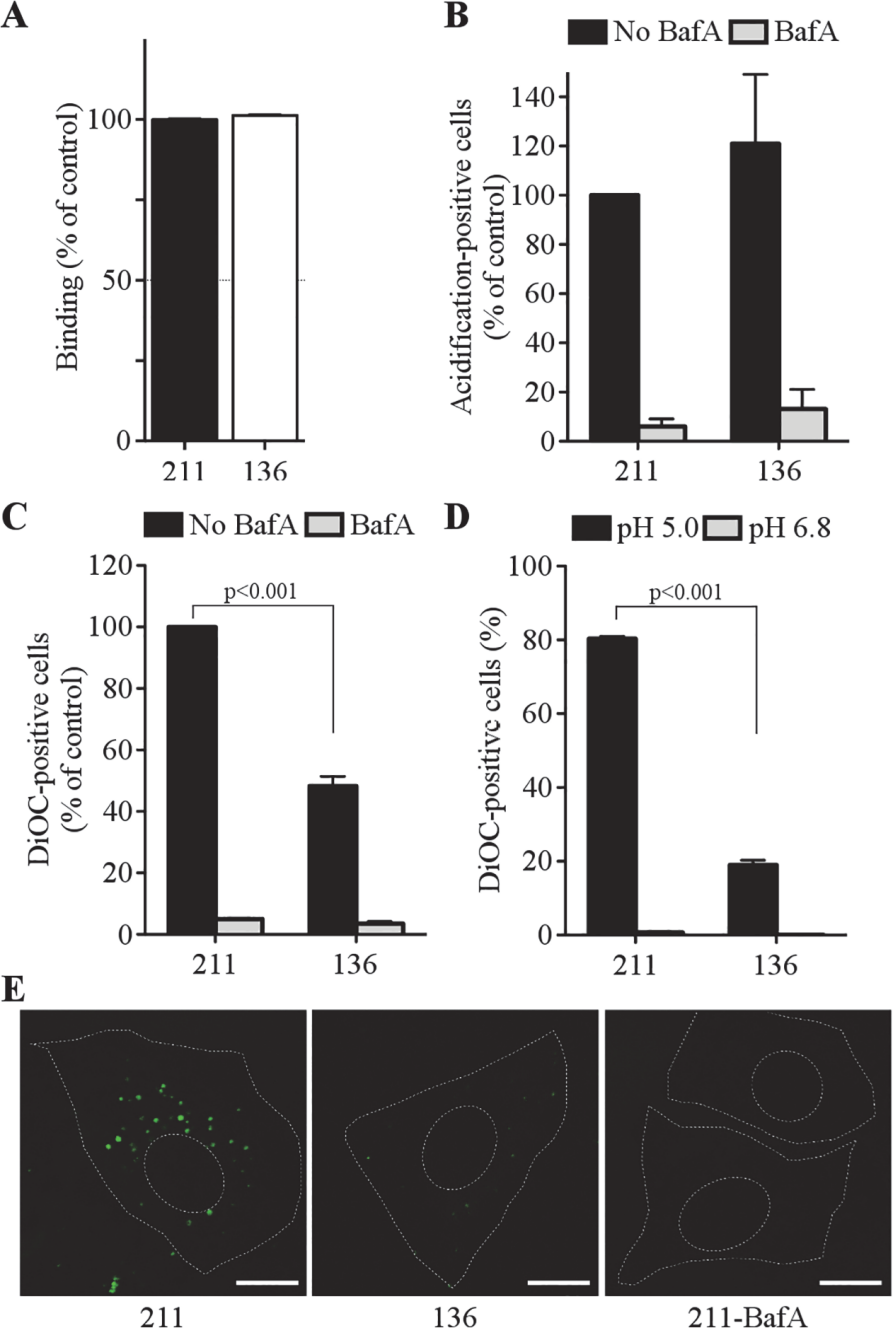
Lipid mixing is blocked in 136 treated X-31 virus in A549 cells. (A) Virus binding to A549 cells. X-31 virus was treated with **211** or **136** and allowed to bind to A549 cells in the cold for 60 min. A549 cells were fixed and stained with a monoclonal HA antibody and analyzed by FACS. (B) X-31 virus was treated with **211** or **136** and allowed to enter A549 cells for 60 min. Cells were fixed and stained for the acidic form of HA. 50 nM Bafilomycin A1 (BafA) was used to block endosome acidification. (C) Lipid mixing in endosomes of A549 cells. X-31 virus was treated with **211** or **136** and allowed to enter cells for 60 min. Cells were fixed and analyzed for DiOC18 dequenching (Student’s t test; p<0.001). (D) Lipid mixing after acid-bypass fusion at the plasma membrane. X-31 virus was treated with **211** or **136**, bound to A549 cells in the cold for 60 min, followed by induction of fusion in warm pH 5.0 (or pH 6.8 for control) medium for 2 min, and fixed. Cells were analyzed for DiOC18 dequenching (Student’s t test; p<0.001). (E) Lipid mixing in endosomes of A549 cells. X-31 virus was treated with **211** or **136** and allowed to enter cells for 60 min. Cells were fixed and analyzed by fluorescence microscopy. The data shown are representative of 3 independent experiments performed in duplicate ± SD. Broken lines depict the outline of cells and nuclei. Bars; 10μm.

To specifically identify the influenza virus entry step **136** inhibits, several experiments were performed in A549 cells as described by Banerjee et. al.[14]. To determine if binding to the host cell was inhibited by **136**, X-31 virus treated with DMSO, **136,** or **211** were bound to cells in the presence of the drug for 1 hour at 4°C so internalization of the virus would not occur. The cells were washed to remove unbound virus, and the bound virus was quantitated using a monoclonal HA antibody and FACS analysis (Fig. 4A). **211** and **136** treated viruses bound to host cells equally, compared to DMSO treatment. Using a low pH conformation specific antibody, **211** or **136** did not prevent the conformational rearrangement of HA in the endosome (Fig. 4B). Addition of Bafilomycin A1, a potent inhibitor of endosomal vacuolar type proton pumps, prevented the low pH conformational change of HA. X-31 viruses labeled with R18 (Octadecyl Rhodamine B Chloride) / SP-DiOC18 (3,3'-Dioctadecyl-5,5'-Di(4-Sulfophenyl)Oxacarbocyanine) was used to study lipid mixing in the endosome (Fig. 4C). **211** did not prevent dequenching of SP-DiOC18, indicating that lipid mixing occurred normally. In contrast, cells infected with **136** treated X-31 virus showed significant decrease in dequenching of SP-DiOC18, as detected by FACS (Fig. 4C) and fluorescence microscopy (Fig. 4E). This indicated that **136** blocks lipid mixing at the endosome. Similar results were obtained when X-31 virus was fused at the plasma membrane using the acid bypass assay (Fig. 4D).

**Fig 5:**
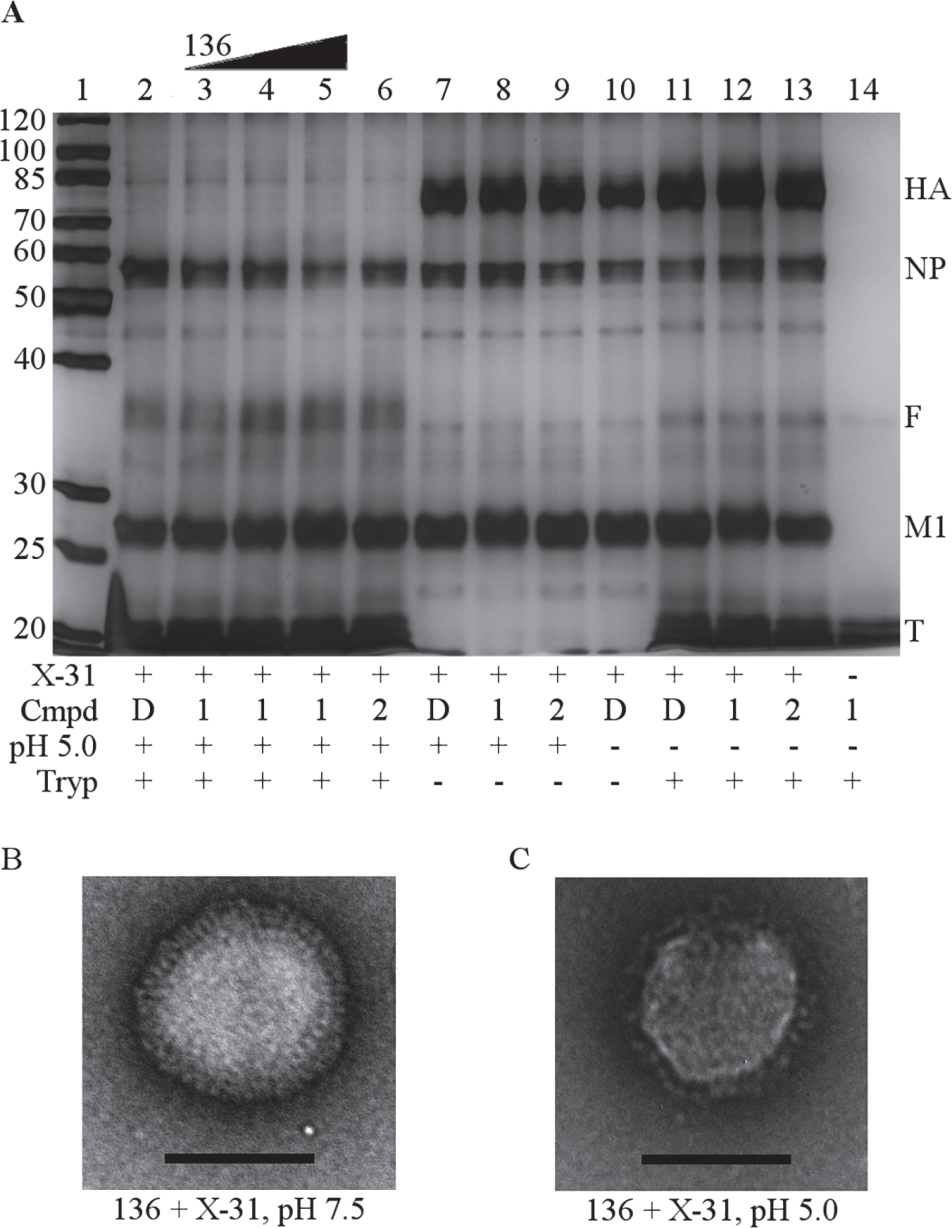
The HA conformational change is not inhibited. (A) X-31 virus was incubated with DMSO (D) 50 nM, 360 nM or 1,060 nM **136** (1) or 1,060 nM **211** (2). Samples were acidified to pH 5.0 and reneutralized (+) or left at pH 7.5 (-). Trypsin (Tryp) was added to cleave unfolded HA (+), or an equal volume of buffer for samples without trypsin was added (-). Trypsin digestion was stopped by addition of AEBSF. The samples were mixed with non-reducing loading buffer and loaded onto a 10% polyacrylamide gel for SDS-PAGE. Lane 1: ladder. Lane 2: HA is degraded by trypsin at pH 5.0. Lanes 3–5: HA is degraded by trypsin at pH 5.0 with **136** treatment, indicating HA is still able to conformationally change. Lane 6: HA is degraded by trypsin at pH 5.0 and **211** treatment. Lanes 7–9: HA is not degraded if trypsin is absent at pH 5.0. Lane 10: HA is not degraded if trypsin is absent at pH 7.5. Lanes 11–13: HA is not degraded by trypsin at neutral pH. Lane 14: trpsin only. 50 nM, 360 nM and 1,060 nM **136** correspond to the EC_50_, EC_90_ and the EC_99_, respectively, for this experiment. Lanes 5, 8, 12, and 14 contain 1,060 nM **136**. HA: hemagglutinin, NP: nucleoprotein, F: fragment, M1: matrix 1 protein, T: trypsin, Cmpd: compound. Representative gel from 3 independent experiments. (B and C) Representative micrographs of X-31 virus treated with **136** are shown. The virus remains intact with organized HA spikes at pH 7.5 (B) but at pH 5.0 the spikes become disorganized consistent with a conformational change (C). Scale bars: 100 nm.

To further confirm that the HA conformational change at low pH is not inhibited by **136**, we performed *in vitro* trypsin susceptibility studies. At a pH of 5.6 or lower, HA unfolds and exposes trypsin sensitive sites of HA that are not present at neutral pH [16]. As seen in Fig. 5A, the controls at pH 5.0 incubated with DMSO (lane 2) or **211** (lane 6) show complete degradation of HA by trypsin and the appearance of an HA fragment band. Acidified viruses treated with increasing concentrations of **136** also show complete degradation of HA by trypsin (lanes 3–5), indicating no inhibition of the HA conformational change. Samples at pH 5.0 but left untreated with trypsin (lanes 7–9) show no degradation of HA and neither does a control sample left at pH 7.5 without trypsin treatment (lane 10). All samples left at pH 7.5 with trypsin treatment (lanes 11–13) show no degradation of HA since trypsin is unable to access the cleavage sites without the conformational change of HA. HA is not destabilized by **136** at pH 7.5. If HA was destabilized, we would expect HA to be degraded at pH 7.5. Additionally, NP and M1 are intact in all samples indicating that pores large enough for trypsin to penetrate into the virus are not present and that the virus remains intact when treated with **136**. Negative stained electron microscopy was used to directly visualize **136** treated X-31 virions at pH 7.5 and 5.0 (Fig. 5B and C). The virions appeared identical to DMSO or **211** treated virions (data not shown). **136** treated virions are intact with organized HA spikes at pH 7.5. At pH 5.0 the virion remains intact and the HA spikes appear more disordered, consistent with a conformational change of HA.

## Discussion

The fusion pathway of influenza virus has been extensively studied but some uncertainties still exist. After binding to cell surface receptors, influenza virus is internalized either by clathrin mediated endocytosis or macropinocytosis [17]. During endosomal maturation, low intraluminal pH triggers an irreversible conformational change of HA, exposing the fusion peptide [18, 19]. The unveiled fusion peptide inserts itself between bilayers of the endosomal membrane and HA refolds into a six-helix bundle, resulting in fusion of the viral membrane to the endosomal membrane [20]. This mechanism is common for all subtypes of influenza A virus and influenza B virus. Our new inhibitor **136** targets this mechanism and has potent antiviral activities against a large variety of influenza viruses, as well as VSV. The results of our time of addition, dilution of inhibitor bound virus, and imaging indicate that the inhibitor prevents viral fusion with cellular membranes. Trypsin sensitivity studies and electron microscopy further revealed that the inhibitor does not perforate the viral envelope, stabilize HA, or prevent the low pH induced conformational change of HA.

*In vitro* studies of **136** treated influenza virus suggests that the structure of the viral envelope was changed by binding of **136** to the virion. The baseline fluorescence of DiD labeled virions was further reduced by treatment with **136** but not **211** or DMSO, suggesting that **136** intercalates in the membrane of the virus in close proximity to DiD (Fig. 3C). Our experiments clearly show that **136** blocks lipid mixing of the influenza virus envelope with the plasma membrane and the late endosomal membrane of A549 cells (Fig. 4C and D). It is possible that integrity of the viral envelope is required for successful viral fusion with cellular membranes. Changes of viral envelope properties induced by **136** may result in the arrest of complete membrane fusion.

A recent paper described the mechanism of inhibition of compound LJ001 which contains a portion structurally similar to **136** [21]. The authors demonstrate that LJ001 and active analogs bind to lipid bilayers [21]. In the presence of light they produce reactive oxygen species that can react with the unsaturated fatty acid chains of phospholipids thus disrupting the biophysical properties of the membrane critical to the fusion process [21]. When **136** in an oxygen atmosphere was subject to UV irradiation for 12 hours, no changes in its NMR profile was observed (S2 Fig.), suggesting high stability of **136**. This result is consistent with **136** tightly binding to the virion membrane. We hypothesize that binding of **136** alters the structure of the viral envelope preventing it from fusing with cellular membranes.

A number of other influenza virus entry inhibitors have been reported (recently reviewed in [22]). Attachment of the virus to the host cell is the first step in the entry pathway. Peptides and small molecules that mimic sialic acid have been developed that bind to the receptor binding pocket of HA thus preventing attachment and internalization of the virus [23–25]. Additionally, small molecules capable of binding to pockets in HA prevent the low pH conformational rearrangement of HA necessary for fusion [26–33]. Compound **136** does not block cell binding (Fig. 4A), or stabilize the low pH form of HA (Fig. 5), but blocks viral entry at the lipid mixing step (Fig. 4C and D).

Current influenza virus inhibitors target viral proteins that are genetically encoded by the virus. However, influenza virus can quickly gain resistant mutations [5, 34, 35]. The optimal strategy would be to target properties of the virus that are not dominantly genetically encoded, reducing the probability of the virus to gain quick resistance through mutation [36]. Rigid amphipathic fusion inhibitors (RAFIs) were developed to inhibit several enveloped viruses by binding to the virion membrane [37]. Resistant mutants of HSV1 could not be generated against RAFIs [37]. Similarly, **136** can inhibit influenza virus as well as VSV by binding to the viral envelope and blocking the virus from fusing with cellular membranes. As with RAFIs, clearly resistant mutants to **136** could not be selected by repeated passages at sublethal concentrations or by selecting a preexisting mutant from a genetically diverse high titer virus stock (data not shown). The binding of **136** to Influenza virions is likely related to the transmembrane domain of HA and the unique lipid composition in the viral envelope, which may still change when substantial mutations occur in viral proteins that determine virus assembly and budding. However, such mutations would take a long period of time to develop and the mutant virus may lose its fitness to become less infectious.

## Supporting Information

S1 Fig The plaque reduction assays of 136 against many different influenza virus strains. Influenza virus plaque reduction assays were performed using monolayers of MDCK-2 cells. Representative data are shown from 3 independent experiments. Data points are the average of 2 replicates ± SD.
(TIF)

S2 Fig 136 NMR profiles. 1-D NMR profiles of **136** before (A) and after (B) 12 hours of UV irradiation and oxygen exposure appear identical, suggesting high stability of **136**.
(TIF)

S1 File Synthesis of compounds 136 and 211. The detailed chemical synthesis protocols for **136** and **211**.
(DOCX)

S1 Table 136 does not alter the pH of virus preparations. The pH of 100 pfu/mL virus preparations. 3 independent experiments are shown.
(TIF)
