# Supplementary figures and images for: Characterization of Potent Fusion Inhibitors of Influenza Virus
Source: PLoS One. 2015 Mar 24;10(3):e0122536. doi: 10.1371/journal.pone.0122536 (PMC4372562; doi:10.1371/journal.pone.0122536)

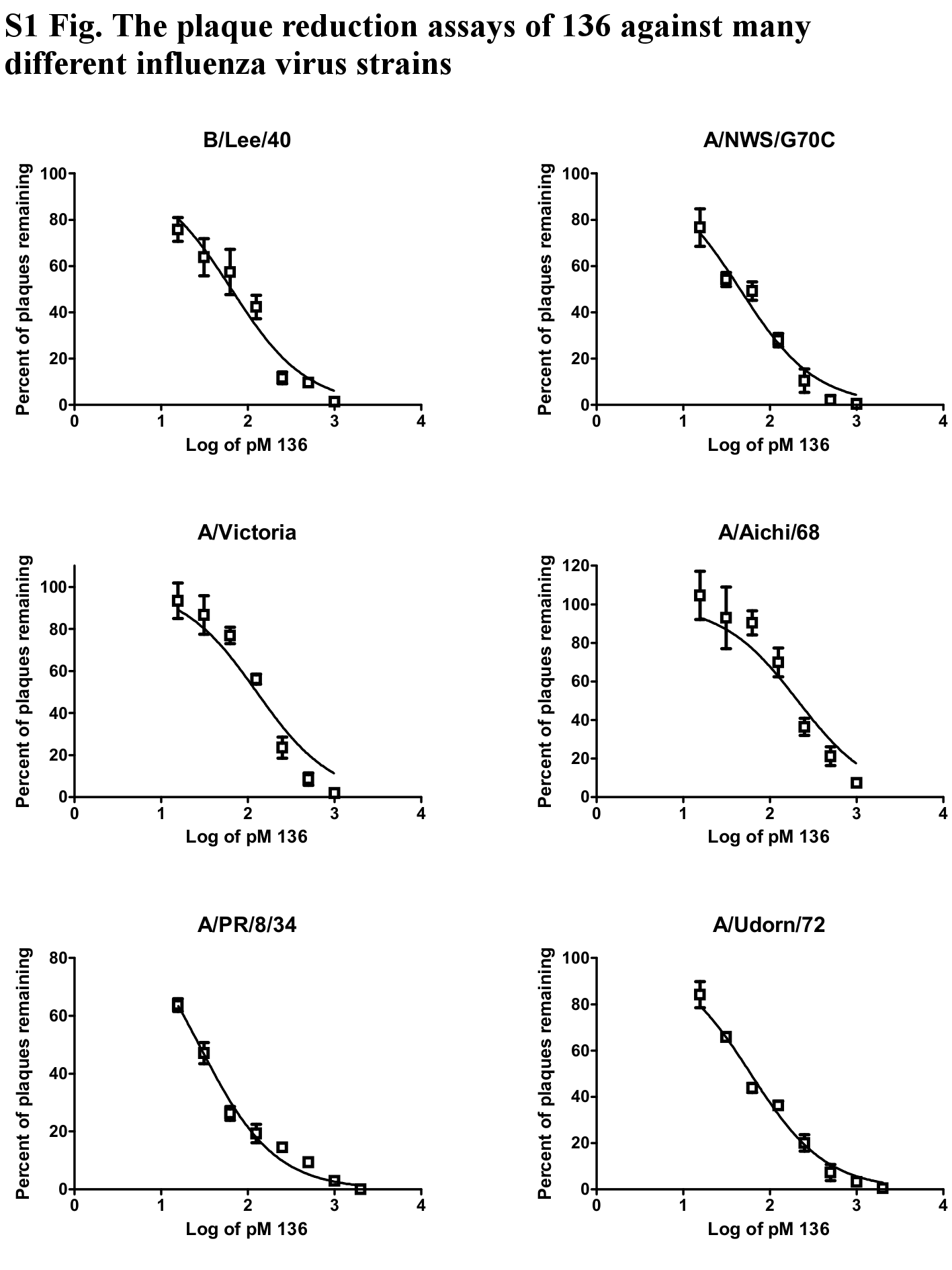

Supplement: S1 Fig — Influenza virus plaque reduction assays were performed using monolayers of MDCK-2 cells. Representative data are shown from 3 independent experiments. Data points are the average of 2 replicates ± SD. (TIF) [file pone.0122536.s001.tif]

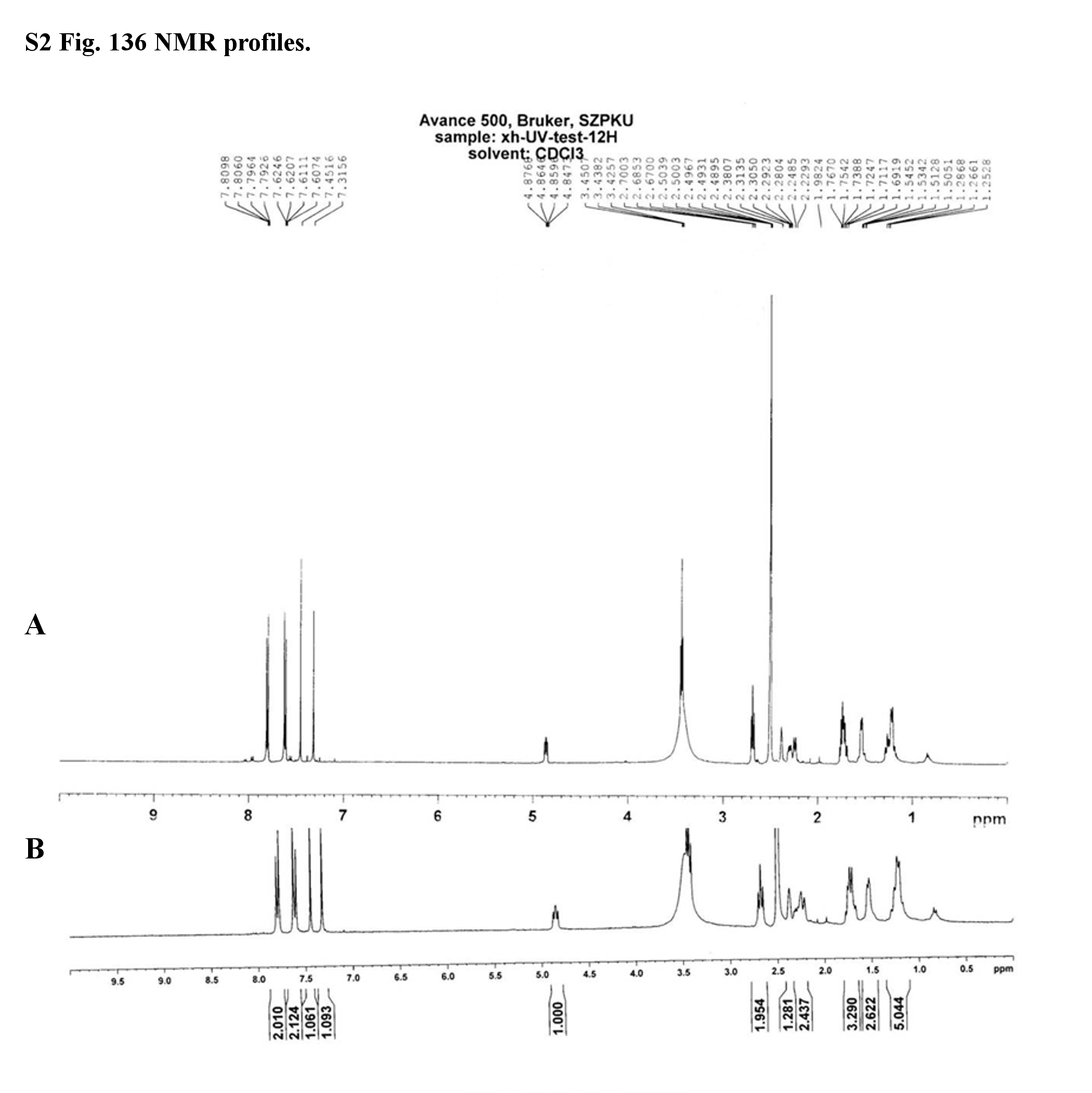

Supplement: S2 Fig — 1-D NMR profiles of 136 before (A) and after (B) 12 hours of UV irradiation and oxygen exposure appear identical, suggesting high stability of 136. (TIF) [file pone.0122536.s002.tif]

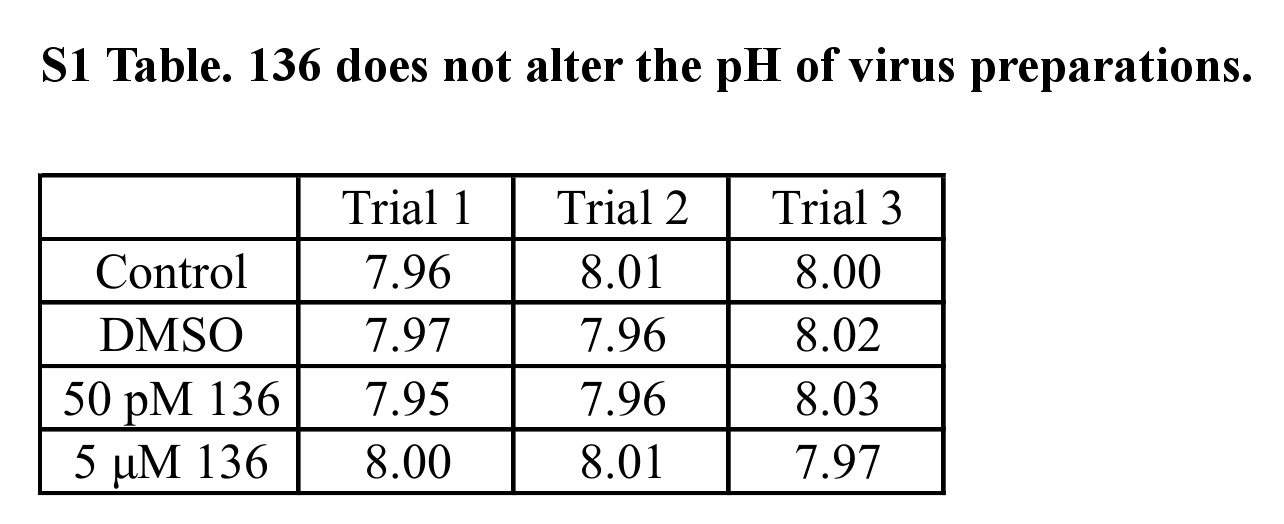

Supplement: S1 Table — The pH of 100 pfu/mL virus preparations. 3 independent experiments are shown. (TIF) [file pone.0122536.s004.tif]
