# Supplementary material for: Characterization of Potent Fusion Inhibitors of Influenza Virus
Source: PLoS One. 2015 Mar 24;10(3):e0122536. doi: 10.1371/journal.pone.0122536 (PMC4372562; doi:10.1371/journal.pone.0122536)

**S1 File. Synthesis of compounds 136 and 211.**

Anhydrous THF and dioxane were distilled from sodium-benzophenone, and dichloromethane was distilled from calcium hydride. All starting materials were purchased from either J&K Chemicals Co. or Sigma Aldrich Chemical Co.. P25H2 was purchased from ChemDiv, Inc. Water was distilled and purified through a Milli-Q water system (Millipore Corp., Bedford, MA). All reactions were carried out under a nitrogen atmosphere with dry solvents under anhydrous conditions, unless otherwise noted. Reactions were monitored by thin-layer chromatography (TLC) carried out on 0.25 mm Tsingdao silica gel plates (60F-254) using UV light as visualizing agent and an ethanolic solution of phosphomolybdic acid and potassium permanganate, and heat as developing agents. Tsingdao silica gel (60, particle size 0.040–0.063 mm) was used for flash column chromatography to purified compounds. Yields refer to chromatographically, unless otherwise stated. All compounds were characterized by ^1^H and ^13^C-NMR using Bruker 300 MHz NMR and/or Bruker 400 MHz NMR spectrometers. Chemical shifts are reported in ppm (δ) relative to the residual solvent peak in the corresponding spectra; chloroform δ 7.26 and δ 75.5, DMSO-*d_6_* δ 2.54 and δ 39.5, and coupling constants (*J*) are reported in hertz (Hz) (where, s *=* singlet, b *=* broad, d *=* doublet, dd *=* double doublet, bd *=* broad doublet, ddd *=* double doublet of dublet, t *=* triplet, td *=* double triplet, q *=* quartet, m *=* multiplet). Mass spectra values are reported as *m/z*.

Abbreviations: NaH = sodium hydride, PPh_3_ = trilphenylphosphine, DIAD = diisopropyl diazene-1,2-dicarboxylate, THF = tetrahydrofuran, DCM = dichloromethane, EA = ethyl acetate, DMF = dimethylformamide.

- General procedure for the synthesis of *(Z)*-(+/-)-*exo*-3-(bicyclo[2.2.1]heptan-2-yl)-5-((5 -(4-chlorophenyl)-3-(5-(piperazin-1-yl)-pentyl)furan-2-yl)methylene)-2-thioxothiazolidin-4- one dihydrochloride **136**:

__

Synthetic route of inhibitor **136**

Synthesis of (+/-)-*exo*-3-(bicyclo[2.2.1]heptan-2-yl)-2-thioxothiazolidin-4-one **3**:

To a solution of triphenylphosphine (6.3 g, 24 mmol) in THF (150 mL) was added DIAD (5.2 g, 24 mmol) at -78 ºC within 2 minutes, and the resultant mixture was stirred at the same temperature for 10 minutes followed by the addition of 1-bicyclo[2.2.1]-heptan-2-ol (mixture of *endo* and *exo,* purchased from Sigma Aldrich Chemical Co., 3.4 g, 30 mmol) at the same temperature. After stirring for 10 minutes rhodanine (2.7 g, 20 mmol) was added to the above solution at -78 ºC, and the resultant mixture was first stirred at -78 ºC for 10 minutes, and then warmed to room temperature, and stirred for 12 h. The reaction was worked up by addition of water (30 mL), and the formed solid was filtered off, and the aqueous phase was extracted with ethyl acetate (3 x 30 mL). The combined organic extracts were washed with brine (20 mL), and dried over anhydrous Na_2_SO_4_. After removal of the solvent under vacuum, the residuce was purified by a flash column chromatography on silica gel (ethyl acetate-hexane) to give (+/-)-*exo*-3-(bicyclo[2.2.1]heptan-2-yl)-2-thioxo- thiazolidin-4-one **3** (3.6 g) in 80% yield as pale yellow solids; ^1^H NMR (300 MHz, CDCl_3_) *δ* 4.78 (td, *J =* 8.4, 6.3 Hz, 1H), 3.84 (s, 1H), 2.46 (s, 1H), 2.42 (s, 1H), 2.25-2.13 (m, 2H), 1.76-1.56 (m, 4H), 1.40-1.10 (m*,* 2H ); HRMS (ESI) calcd for C_10_H_14_NOS_2_ [M + H]^+^ 228.0517, found 228.0510.

Synthesis of 2-(4-chlorophenyl)-4-iodofuran **6**:

To a stirred solution of THP-protected propargylic alcohol **4** (16.8 g, 0.12 mol) in dry THF (100 mL) was added *n*-BuLi (0.12 mol, 2.5 M in hexane) at -78^o^C, and the mixture was stirred for 30 min at -30 ^o^C, followed by addition of the aldehyde **5** (14.0 g, 0.10 mol) in THF (50 mL) at -78 ^o^C. The mixture was stirred for 30 min at -78^o^C, followed by reaction at 0 ^o^C for 30 min at 0 ^o^C.The reaction was worked up by addition of a saturated ice/water solution of NH_4_Cl (100 mL), followed by Et_2_O (100 mL) and the mixture was extracted with ethyl acetate (3 x 50 mL). The combined organic extracts were washed with saturated brine, and then dried with MgSO_4_. The solvent was removed under vacuum, and the residue was dissolved in DCM (50 mL). To this solution was added to a solution of a solution of active MnO_2_ (3.0 mol) in DCM (100 mL) at room temperature, and the mixture was stirred for 12 h. The reaction was worked up by filtration, and the filtrate was dried with MgSO_4_.

The solvent was removed under vacuum, and the residue was redissolved in methanol (100 mL). To this solution was added sodium iodide (75.0 g, 0.50 mol), *p*-toluenesulfonic acid monohydrate (19.0 g, 0.10 mol) at room temperature, and the reaction mixture was stirred at the same temperature for 2 h. The reaction mixture was worked up by addition of a saturated solution of NaHCO_3_ and Na_2_SO_3_, and the resultant mixture was extracted with CH_2_C1_2_. The combined organic extracts were dried with MgSO_4_.The solvent was concentrated under vacuum,and the residue was purified by a flash chromatography on neutral aluminium oxide (hexane/ethyl acetate) to give 4-iodo-2-(4-chlorophenyl)-4-iodofuran **6** (18.2 g, 60%) as pale yellow solid. ^1^H NMR (300 MHz, CDCl_3_) *δ* 7.56 (d, *J*= 13 Hz, 2H), 7.46 (s, 1H), 7.35 (d, *J*= 11 Hz, 2H), 6.71 (s, 1H); HRMS (ESI) calcd for C_10_H_7_ClIO [M + H]^+^ 304.9230, found 304.9228.

Synthesis of 5-(5-(4-chlorophenyl)furan-3-yl)pent-4-yn-1-ol **7**:

To a solution of 2-(4-chlorophenyl)-4-iodofuran **6** (3.04 g, 10.0 mmol) and pent-4-yn-1-ol (1.68 g, 20.0 mmol) in degassed THF (80 mL) was sequentially added Pd(PPh_3_)Cl_2_ (0.70 g, 1.0 mmol), CuI (0.38 g, 2.0 mmol) and Et_3_N (5.05g, 50.0 mmol), andthe reaction mixture was stirred for 10 h under refluxing conditions. The reaction was worked up by removal of the solvent under vacuum, and the residue was purified by a flash chromatography (hexane/ethyl acetate) on silica gel to give 5-(5-(4-chlorophenyl)-furan-3-yl)pent-4-yn-1-ol **7** (2.47 g, 95%) as yellow oil. ^1^H NMR (300 MHz, CDCl_3_) *δ* 7.55 (d, *J*= 6.5 Hz, 2H), 7.33 (d, *J*= 6.5 Hz, 2H), 7.26 (s, 1H), 6.53 (d, *J*= 1.8 Hz, 1H), 3.72 –3.69 (m, 2H), 2.56 –2.53 (m, 2H), 1.89-1.82 (m, 2H), 1.67 (board, 1H); HRMS (ESI) calcd for C_15_H_14_ClO_2_ [M + H]^+^ 261.0682, found 261.0680.

Synthesis of 5-(5-(4-chlorophenyl)furan-3-yl)pentan-1-ol **8**:

To a solution of 5-(5-(4-chlorophenyl)furan-3-yl)pent-4-yn-1-ol **7** (2.00 g, 7.7 mmol) in EtOH (50 mL) was added 10% Pd/C (0.20 g), and the mixture was stirred under a balloon pressure of hydrogen for 12 h.. The reaction was worked up by filtration of the reaction mixture, and the filtrate was concentrated, and the residue was purified by a flash chromatography (hexane/ethyl acetate) on silica gel to give 5-(5-(4-chlorophenyl)furan-3- yl)pentan-1-ol **8** (2.03 g, ~100%) as the pale yellow oil. ^1^H NMR (300 MHz, CDCl_3_) *δ* 7.55 (d, *J*= 8.7 Hz, 2H), 7.33 (d, *J*= 8.7 Hz, 2H), 7.23 (d, *J*= 0.9 Hz, 1H), 6.53 (s, 1H), 3.66 (t, *J* = 6.0 Hz, 2H), 2.45 (t, *J* =7.5 Hz, 2H), 1.65-1.56 (m, 6H), 1.49-1.40 (m, 2H); HRMS (ESI) calcd for C_15_H_18_ClO_2_ [M + H]^+^ 265.0995, found 265.0999.

Synthesis of 5-(5-(4-chlorophenyl)-2-formylfuran-3-yl)pentyl acetate **9**:

To a solution of 5-(5-(4-chlorophenyl)furan-3-yl)pentan-1-ol **8** (2.03 g, 7.7 mmol) in DCM (50 mL) was added Ac_2_O (3.11 g, 30.8 mmol) and DMAP (0.10 g, 0.8 mmol) at room temperature, and the mixture was stirred at the same temperature for 2h. The reaction was quenched by addition of H_2_O (50 mL), and the mixture was extracted by ethyl acetate (3 x 50 mL). The combined organic extracts were washed with saturated NaHCO_3_, and dried with MgSO_4_, the solvent was removed under vacuum, and the residue was purified by a flash chromatography (hexane/ethyl acetate) on silica gel to give 2.35 g of 5-(5-(4-chlorophenyl)furan-3-yl)pentyl acetate as the pale yellow oil in the quantitative yield. To a solution of 5-(5-(4-chlorophenyl)furan-3-yl)pentyl acetate (1.53 g, 5.0 mmol) in *N,N*-dimethyl formamide (25 mL) was added a solution of *N,N*-dimethyl formamide (10 mL) and phosphorus oxychloride (0.92 g, 6.0 mmol) at 0°C under nitrogen, and the resultant mixture was first stirred at to room temperature for 30 min, and then at 60°C for 2 h. The reaction was worked by addition of a saturated solution of Na_2_CO_3_ at 0 °C to neutralize topH 6, and the mixture was extracted with ethyl acetate twice. The combined organic extracts was washed with saturated NaHCO_3_, and then dried with MgSO_4_,.The solvent was removed under vacuum, and the residue was purified by a flash chromatography (hexane/ethyl acetate) on silica gel to give 5-(5-(4-chlorophenyl)- 2-formylfuran-3-yl)pentyl acetate **9** (1.34 g, 80% in two steps) as pale yellow oil. ^1^H NMR (300 MHz, CDCl_3_) *δ* 9.76 (s, 1H), 7.72 (d, *J*= 8.7 Hz, 2H), 7.41 (d, *J*= 11 Hz, 2H), 6.70 (s, 1H), 4.06 (t, *J* = 6.0 Hz, 2H), 2.84 (t, *J* = 6.0 Hz, 2H), 2.03 (s, 3H), 1.72-1.63 (m, 4H), 1.49-1.41 (m, 2H); HRMS (ESI) calcd for C_18_H_20_ClO_4_ [M + H]^+^ 335.1050, found 335.1043.

Synthesis of 5-(4-chlorophenyl)-3-(5-hydroxypentyl)furan-2-carbaldehyde **10**:

To the solution of 5-(5-(4-chlorophenyl)-2-formylfuran-3-yl)pentyl acetate **9** (1.0 g, 3.0 mmol) in MeOH was added K_2_CO_3_ (2.07 g, 15.0 mmol), the suspension was stirred at room temperature until **9** was fully consumed (1 h). The reaction was worked up by addition of H_2_O, the resultant mixture was extracted with EA. The combined organic phases were concentrated; the residue was purified by a flash chromatography (hexane/ethyl acetate) on silica gel to give 5-(4-chlorophenyl)- 3-(5-hydroxypentyl)furan-2-carbaldehyde **10** (0.88 g, ~100%) as pale yellow oil. ^1^H NMR (300 MHz, CDCl_3_) *δ* 9.73 (s, 1H), 7.72 (d, *J*= 13 Hz, 2H), 7.37 (d, *J*= 13 Hz, 2H),6.69 (s, 1H), 3.64 (t, *J* = 6.3 Hz, 2H), 2.81 (t, *J* =7.2 Hz, 2H), 1.89 (broad, 1H), 1.71-1.56 (m, 4H), 1.49-1.41 (m, 2H); HRMS (ESI) calcd for C_16_H_18_ClO_3_ [M + H]^+^ 293.0944, found 293.0937.

Synthesis of *(Z)*-(+/-)-*exo*-3-(bicyclo[2.2.1]heptan-2-yl)-5-((5-(4-chlorophenyl)-3-(5-hydroxy- pentyl)- furan-2-yl)methylene)-2-thioxothiazolidin-4-one **11**:

To a solution of (+/-)-*exo*-3-(bicyclo[2.2.1]heptan-2-yl)-2-thioxothiazolidin-4-one **3** (0.14 g, 0.6 mmol) and 5-(4-chlorophenyl)- 3-(5-hydroxypentyl)furan-2-carbaldehyde **10** (0.15 g, 0.5 mmol) in EtOH (5 mL) was added a catalytic amount of anhydrous piperidine (two drops) at room temperature, and the mixture was stirred for 12 h. The reaction was worked up by addition of ethyl acetate (50 mL), and the combined organic extracts were washed with water (3 x 10 mL), and then dried over anhydrous Na_2_SO_4_. The solvent was removed under vacuum, and the residue was purified by a flash chromatography (ethyl acetate/hexane) to afford *(Z)*-(+/-)-*exo*-3-(bicyclo[2.2.1]heptan-2-yl)-5-((5-(4-chlorophenyl)-3-(5-hydroxylpentyl)furan-2-yl)-mthylene)-2-thioxothiazolidin-4-one **11** (0.19 g, 75%) as red solid. ^1^H NMR (300 MHz, CDCl_3_) *δ* 7.65 (d, *J*= 8.7 Hz, 2H), 7.43 (d, *J*= 8.7 Hz, 2H), 7.38 (s, 1H), 6.72 (s, 1H), 4.97 (td, *J =* 8.4, 6.0 Hz, 1H), 3.66 (t, *J* = 6.3 Hz, 2H), 2.62 (t, *J* = 7.2 Hz, 2H), 2.55 (s, 1H), 2.46 (s, 1H), 2.40-2.23 (m, 2H), 1.75 (t, *J =* 7.3, 2.4 Hz, 1H), 1.76-1.63 (m*,* 3H), 1.60-1.40 (m*,* 4H ); HRMS (ESI) calcd for C_26_H_29_ClNO_3_S_2_ [M + H]^+^ 502.1277, found 502.1265.

Synthesis of *(Z)*-(+/-)-*exo*-3-(bicyclo[2.2.1]heptan-2-yl)-5-((5-(4-chlorophenyl)-3-(5-iodo- pentyl)furan-2-yl)-methylene)-2-thioxothiazolidin-4-one **12**:

To a solution of *(Z)*-(+/-)-*exo*-3-(bicyclo[2.2.1]heptan-2-yl)-5-((5-(4-chlorophenyl)-3- (5-hydroxy-pentyl)furan-2-yl)methylene)-2-thioxothiazolidin-4-one **11** (0.15 g, 0.30 mmol), PPh_3_ (0.12 g, 0.46 mmol) and imidazole (0.04 g, 0.60 mmol) in THF (60 mL) was added I_2_ (0.10 g, 0.40 mmol), the mixture was stirred at room temperature for 2h. The solvent were removed, and the residue was extracted with EA, and the combined organic extracts were washed with a saturated solution of Na_2_S_2_O_3_ and then dried MgSO_4_. The solvent was removed under vacuum, and the residue was purified by a flash chromatography (ethyl acetate/hexane) on silica gel to give *(Z)*-(+/-)-*exo*-3-(bicyclo[2.2.1]heptan-2-yl)-5-((5-(4- chlorophenyl)-3-(5-iodopentyl)furan-2-yl)-methylene)-2-thioxothiazolidin-4-one **12** (0.13 g, 70%) as yellow oil. ^1^H NMR (300 MHz, CDCl_3_) *δ* 7.67 (d, *J*= 11 Hz, 1H), 7.43 (d, *J*= 8.7 Hz, 1H), 7.36 (s, 1H), 6.73 (s, 1H), 6.71 (d, *J =* 3.6 Hz, 1H), 4.97 (td, *J =* 8.7, 6.0 Hz, 1H), 2.86 (t, *J* = 7.2 Hz, 2H), 2.67 (t, *J* = 7.2 Hz, 2H), 2.46 (s, 1H), 2.40-2.23 (m, 2H), 1.82 (dt, *J =* 7.3, 2.4 Hz, 1H), 1.76-1.63 (m*,* 3H), 1.60-1.40 (m*,* 4H ); HRMS (ESI) calcd for C_26_H_28_ClINO_2_S_2_ [M + H]^+^ 612.0295, found 612.0285.

Synthesis of *(Z)*-(+/-)-*exo*-*tert*-butyl 4-(5-(2-((3-(bicyclo[2.2.1]heptan-2-yl)-4-oxo-2-thioxo- thiazolidin-5-ylidene)methyl)-5-(4-chlorophenyl)furan-3-yl)pentyl)piperazine-1-carboxylate **13**:

To a solution of *(Z)*-(+/-)-*exo*-3-(bicyclo[2.2.1]heptan-2-yl)-5-((5-(4-chlorophenyl)-3-(5 -iodopentyl)furan-2-yl)methylene)-2-thioxothiazolidin-4-one **12** (0.12 g, 0.20 mmol) in dry DCM (10 mL) was added *tert*-butyl piperazine-1-carboxylate (0.08 g, 0.43 mmol), and the mixture was stirred at room temperature for 12 h. The solvent was removed, and the residue was purified by a flash chromatography (ethyl acetate/hexane) on silica gel to give *(Z)*-(+/-)-*exo*-*tert*-butyl 4-(5-(2-((3-(bicyclo[2.2.1]heptan-2-yl)-4-oxo-2-thioxothiazolidin-5- ylidene)-methyl)-5-(4-chlorophenyl)furan-3-yl)pentyl)piperazine-1-carboxylate **13** (0.12 g, 90%) as red solid. ^1^H NMR (300 MHz, CDCl_3_) *δ* 7.70 (d, *J =* 8.7 Hz, 2H), 7.45 (d, *J =* 8.4 Hz, 2H), 7.39 (s*,* 1H), 6.74 (s*,* 1H), 4.90 (dd, *J =* 8.7, 6.3 Hz, 1H), 3.45 (broad, 4H), 2.64-2.57 (m, 3H), 2.48-2.30 (m, 10H), 1.90-1.53 (m, 7H), 1.50 (s, 9H), 1.48-1.20 (m, 6H); HRMS (ESI) calcd for C_35_H_45_ClN_3_O_4_S_2_ [M + H]^+^ 670.2540, found 670.2542.

Synthesis of *(Z)*-(+/-)-*exo*-3-(bicyclo[2.2.1]heptan-2-yl)-5-((5-(4-chlorophenyl)-3-(5- (piperazin-1-yl)-pentyl)furan-2-yl)methylene)-2-thioxothiazolidin-4-one dihydrochloride **136**:

*(Z)*-(+/-)-*exo*-*tert*-butyl 4-(5-(2-((3-(bicyclo[2.2.1]heptan-2-yl)-4-oxo-2-thioxothiazolidin -5-ylidene)- methyl)-5-(4-chlorophenyl)furan-3-yl)pentyl)piperazine-1-carboxylate **13** (0.12 g, 0.18 mmol) was mixed with a saturated solution of HCl (gas) in methanol (20 mL) at 0 ^o^C, and the resultant mixture was stirred at room temperature for 2 h. The reaction was worked up by removal of the organic solvent under vacuum, and the residue was purified by a recrystallization from diethyl ether to give *(Z)*-(+/-)-*exo*-3-(bicyclo[2.2.1]heptan-2-yl)-5- ((5-(4-chlorophenyl)-3-(5-(piperazin-1-yl)-pentyl)furan-2-yl)methylene)-2-thioxothiazolidin-4-one dihydrochloride **136** (0.12 g, ~100%) as reddish brown solid. ^1^H NMR (300 MHz, DMSO-*d_6_*) *δ* 11.8 (board, 1H), 9.72 (board, 2H), 7.81 (d, *J =* 9.0 Hz, 2H), 7.65 (d, *J =* 6.0 Hz, 2H), 7.46 (s*,* 1H), 7.37 (s*,* 1H), 4.85 (t, *J =* 6.0 Hz, 1H), 3.56-3.53 (m, 2H), 3.48-3.39 (m, 4H), 3.26-3.19 (m*,* 2H), 3.35-3.12 (m*,* 4H), 2.66 (t, *J =* 7.2 Hz, 2H), 2.37 (s, 1H), 2.28-2.19 (m*,* 2H), 1.74-1.63 (m*,* 4H), 1.54-1.53 (m*,* 2H), 1.40-1.36  (m*,* 2H), 1.25-1.20 (m*,* 3H); ^13^C NMR (75.5 MHz, DMSO-*d_6_*) *δ* 196.1, 167.1, 156.3, 146.2, 137.9, 133.9, 129.6, 127.5, 126.2, 117.4, 115.5, 111.9, 62.4, 55.6, 47.9, 37.8, 36.3, 35.3, 29.3, 28.0, 26.0, 24.6, 23.0; HRMS (ESI) calcd for C_30_H_39_Cl_3_N_3_O_2_S_2_ [M – 2HCl + H]^+^ 570.2016, found 570.1943.

- General procedure for the synthesis of (*E*)- *endo/exo*-1-(bicyclo[2.2.1]heptan-2-yl)-3- ((5-(4-chlorophenyl)-3-(5-hydroxy-pentyl)- furan-2-yl)methylene)pyrrolidine-2,5-dione **211**:

Synthetic route of inhibitor **211**

Synthesis of *endo/exo*-1-(bicyclo[2.2.1]heptan-2-yl)-1H-pyrrole-2,5-dione **16**:

To a solution of 1-bicyclo[2.2.1]heptan-2-amine **14** (3.36 g, 30.0 mmol, mixture of *endo* and *exo*, purchased from J&K Chemicals Co.) and furan-2,5-dione **15** (1.98 g, 20.0 mmol) in toluene (150 mL) was added H_2_SO_4_ (5.0 mL) and Et_3_N (5.0 mL) and the mixture was stirred under refluxing conditions for 12h. After removal of the solvents, the residue was resolved in ethyl acetate (150 mL), and the organic phase was washed sequentially with H_2_O, NaHCO_3_, and brine (20 mL), and finally dried over anhydrous Na_2_SO_4_. After removal of the solvent under vacuum, the crude product was purified by a flash column chromatography on silica gel (ethyl acetate/hexane) to afford *endo/exo*-1-(bicyclo[2.2.1]heptan-2-yl)-1H-pyrrole-2,5-dione **16** (2.51 g, *endo*:*exo =* 5:2, 65%) as whit solid. ^1^H NMR (300 MHz, CDCl_3_) *δ* 6.76 (s, 1H), 4.31-4.10 (m*,* 0.75H), 4.00-3.85 (m*,* 0.25H), 2.52-2.05 (m, 4H), 1.62-1.50 (m*,* 3H), 1.40-1.25 (m*,* 3H ); HRMS (ESI) calcd for C_11_H_13_NO_2_Na [M + Na]^+^ 214.0844, found 214.0838.

Synthesis of (*E*)-*endo/exo*-1-(bicyclo[2.2.1]heptan-2-yl)-3-((5-(4-chlorophenyl)-3-(5- hydroxylpentyl)-furan-2-yl)methylene)pyrrolidine-2,5-dione **211**:

To a solution of 5-(4-chlorophenyl)-3-(5-hydroxypentyl)furan-2-carb-aldehyde **10** (1.46 g, 5.0 mmol) and *endo/exo*-1-(bicyclo[2.2.1]heptan-2-yl)-1H-pyrrole-2,5-dione **16** (0.96 g, *endo*:*exo =* 5:2, 5.0 mmol) in dry THF (50 mL) was added PPh_3_ (1.31 g, 5.0 mmol), and the mixture was stirred under refluxing conditions for 12h. The reaction was worked up by removal of the solvents, and the residue was resolved in ethyl acetate (150 mL), then washed with brine (20 mL), and finally dried over anhydrous Na_2_SO_4_. The solvent was removed under vacuum, and the residue was purified by a flash chromatography (ethyl acetate/hexane) on silica gel to give (*E*)-*endo/exo*-1-(bicyclo[2.2.1]-heptan-2-yl)-3-((5-(4-chlorophenyl) -3-(5-hydroxypentyl)furan-2-yl)- methylene)pyrrolidine-2,5-dione **211** (1.05 g, *end*:*exo =* 5:2, 45%) as yellow solid. ^1^H NMR (400 MHz, CDCl_3_) *δ* 7.60 (d, *J =* 8.6 Hz, 2H), 7.41 (d, *J =* 8.4 Hz, 2H), 7.37-7.34 (m*,* 1H), 6.70 (s*,* 1H), 4.50-4.39 (m*,* 0.75H), 4.15-4.05 (m*,* 0.25H), 3.73 (d, *J =* 2.0 Hz, 1H), 3.70-3.65 (m, 2H), 2.62 (td, *J =* 7.2, 3.2 Hz, 2H), 2.54 (s, 1H), 2.39 (s, 1.4H), 2.30-2.20 (m, 0.75H), 1.90-1.75 (m, 3H), 1.54-1.20 (m, 10H); ^13^C NMR (100 MHz, CDCl_3_) *δ* 167.96, 156.73, 146.46, 137.11, 134.88, 129.32, 127.37, 125.80, 118.13, 115.15, 110.00, 62.63, 41.40, 37.93, 36.48, 35.49, 32.33, 30.04, 29.46, 27.90, 25.35, 25.15; HRMS (ESI) calcd for C_27_H_30_ClNO_4_Na [M + Na]^+^ 490.1761, found 490.1754.

**Spectrum data of compounds 136 and 211:**


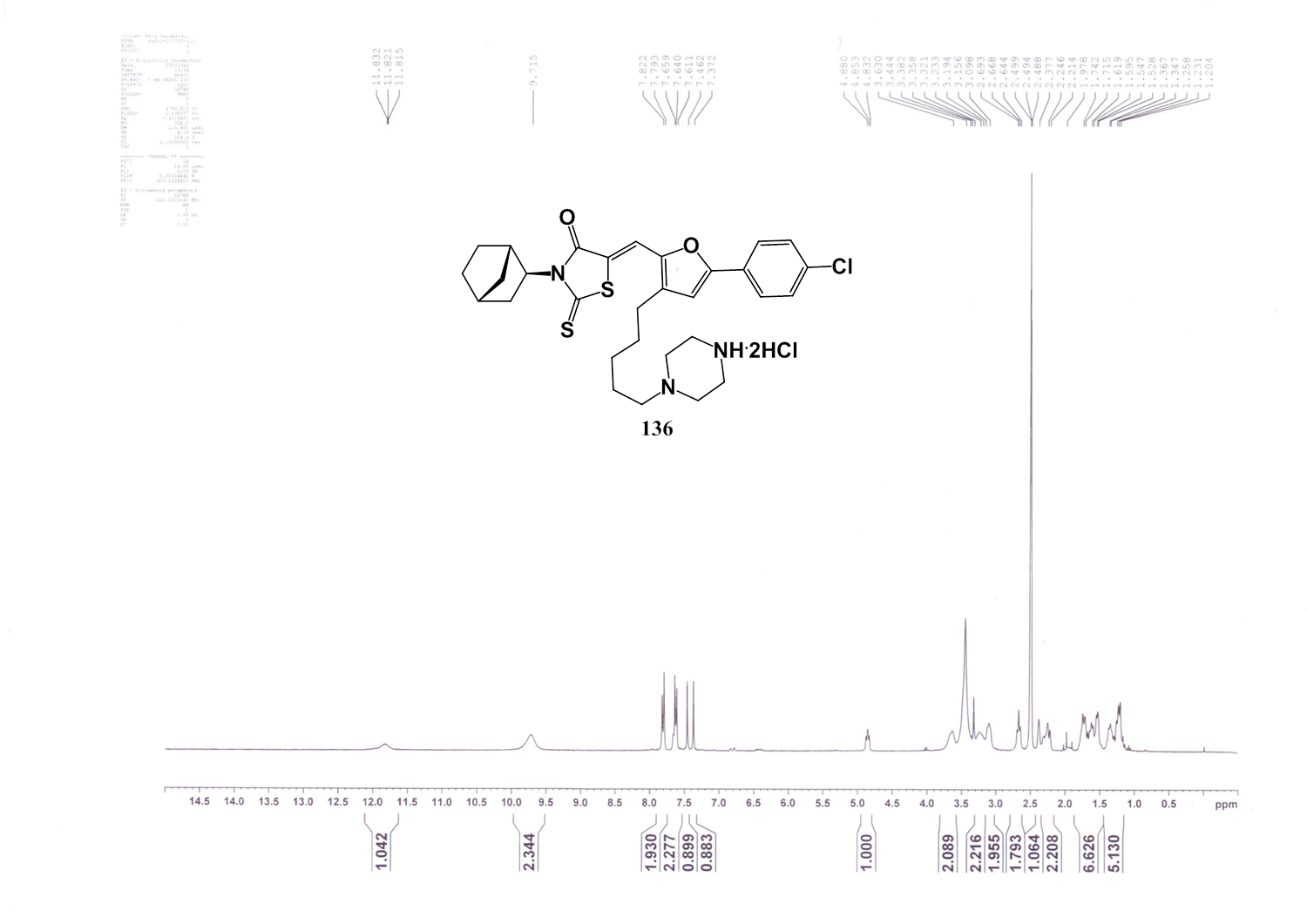

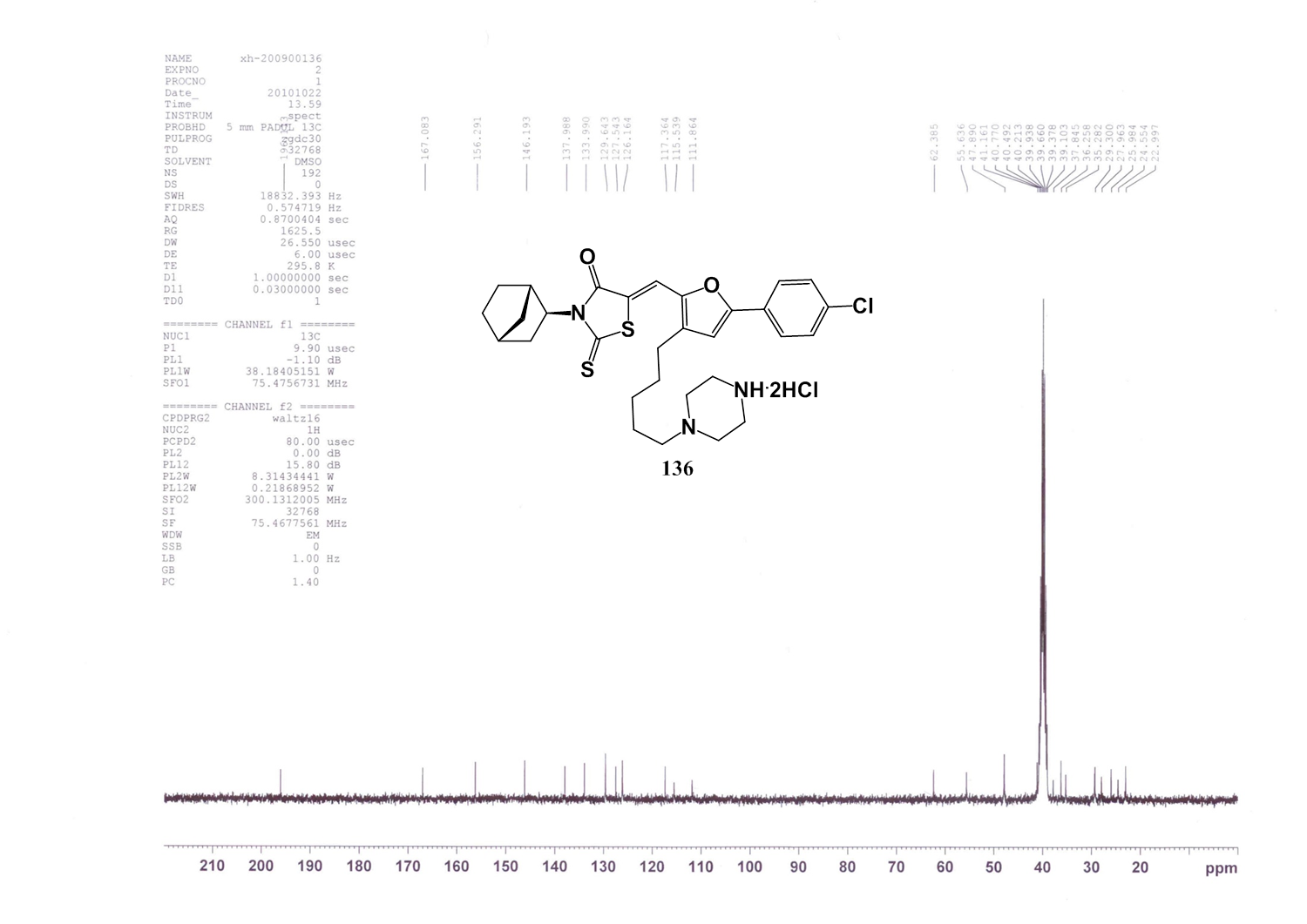

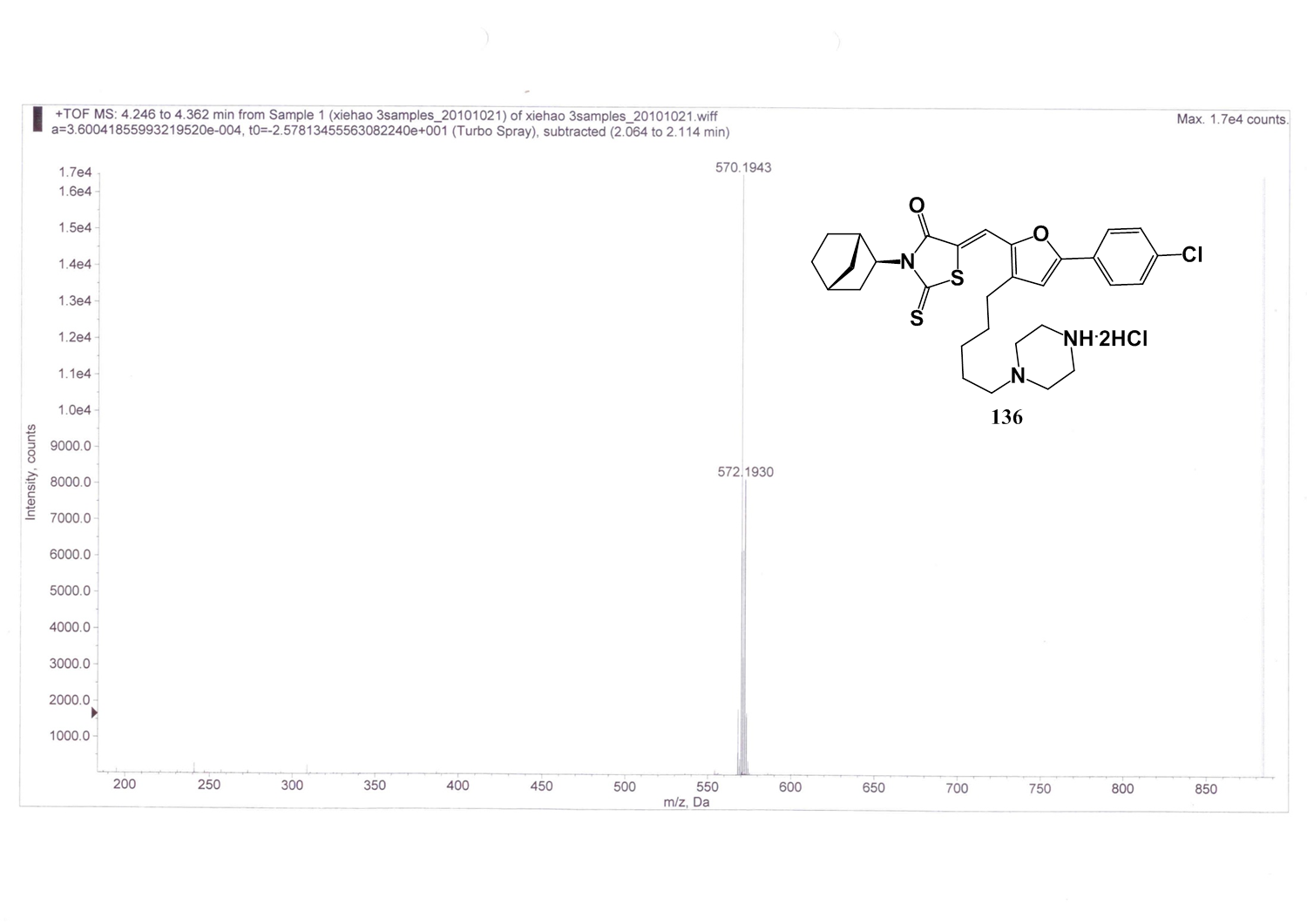

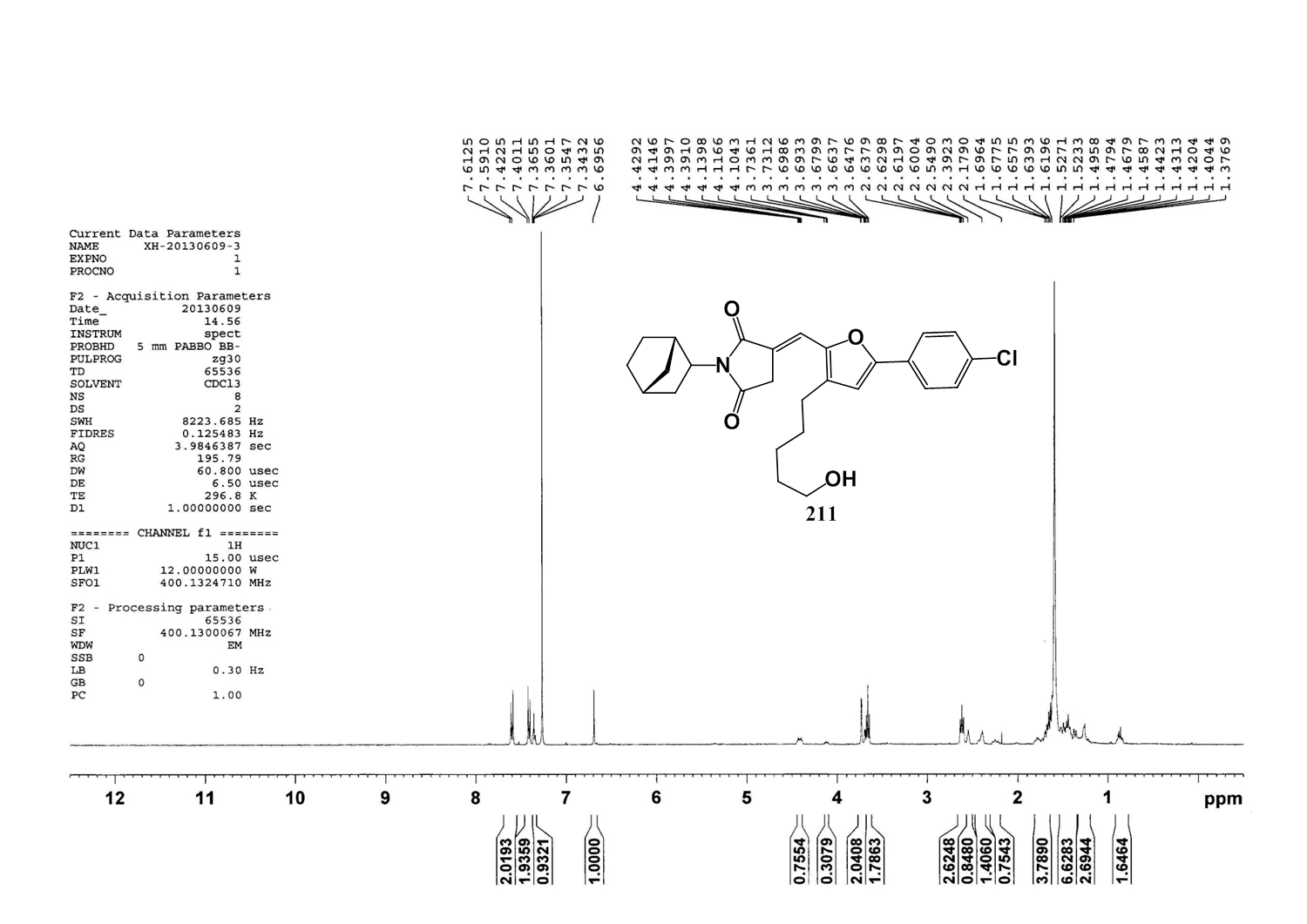

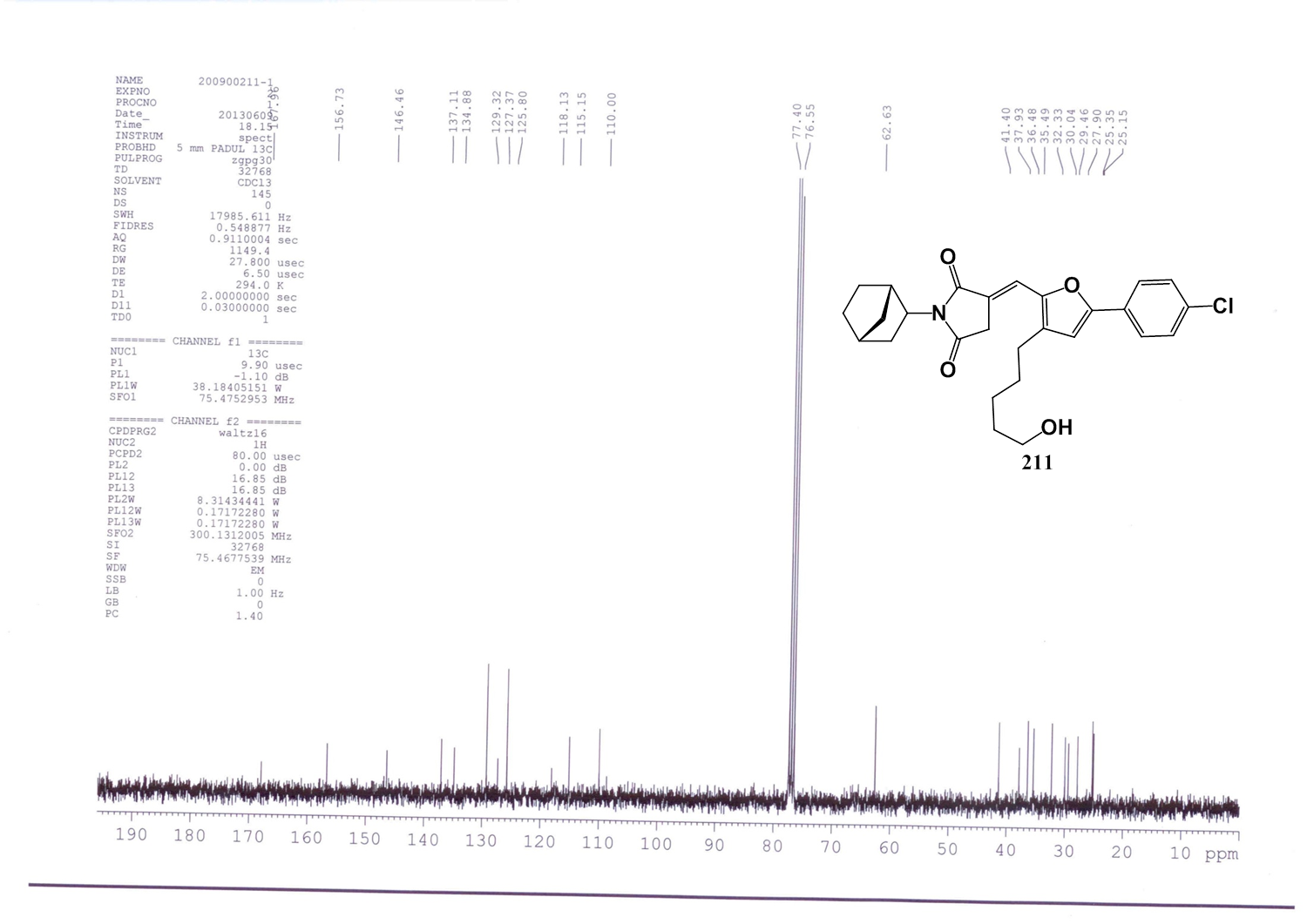

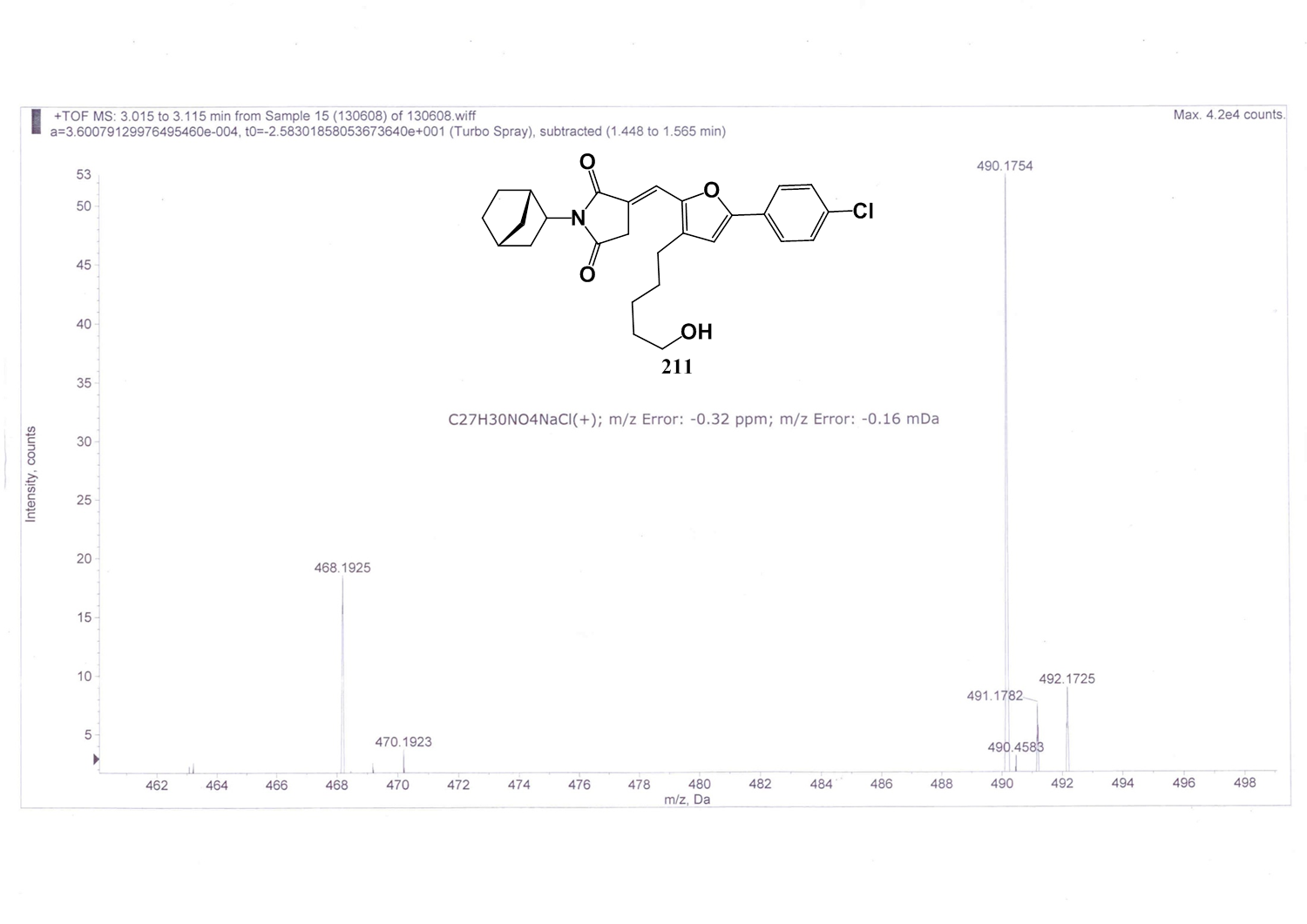

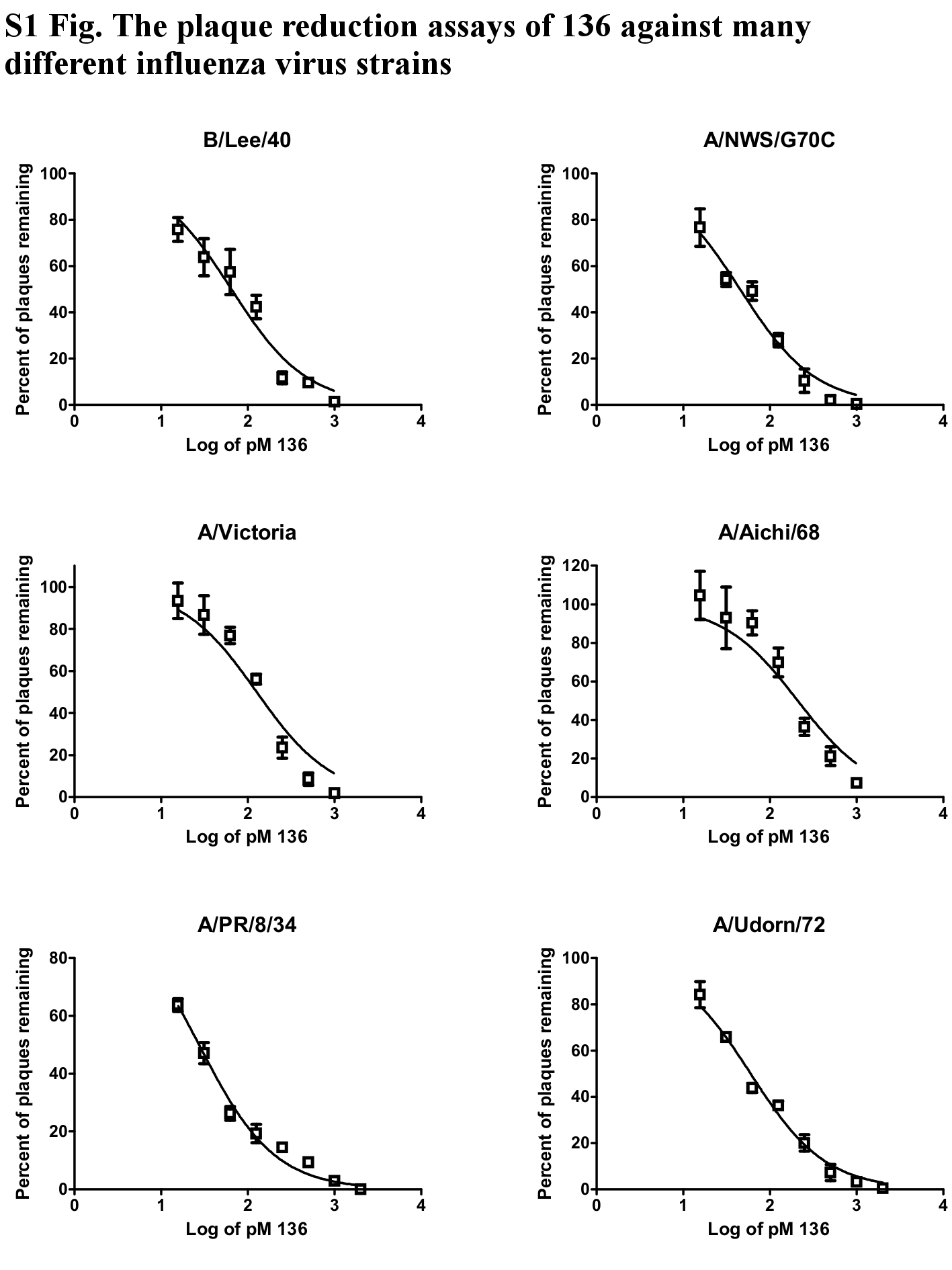

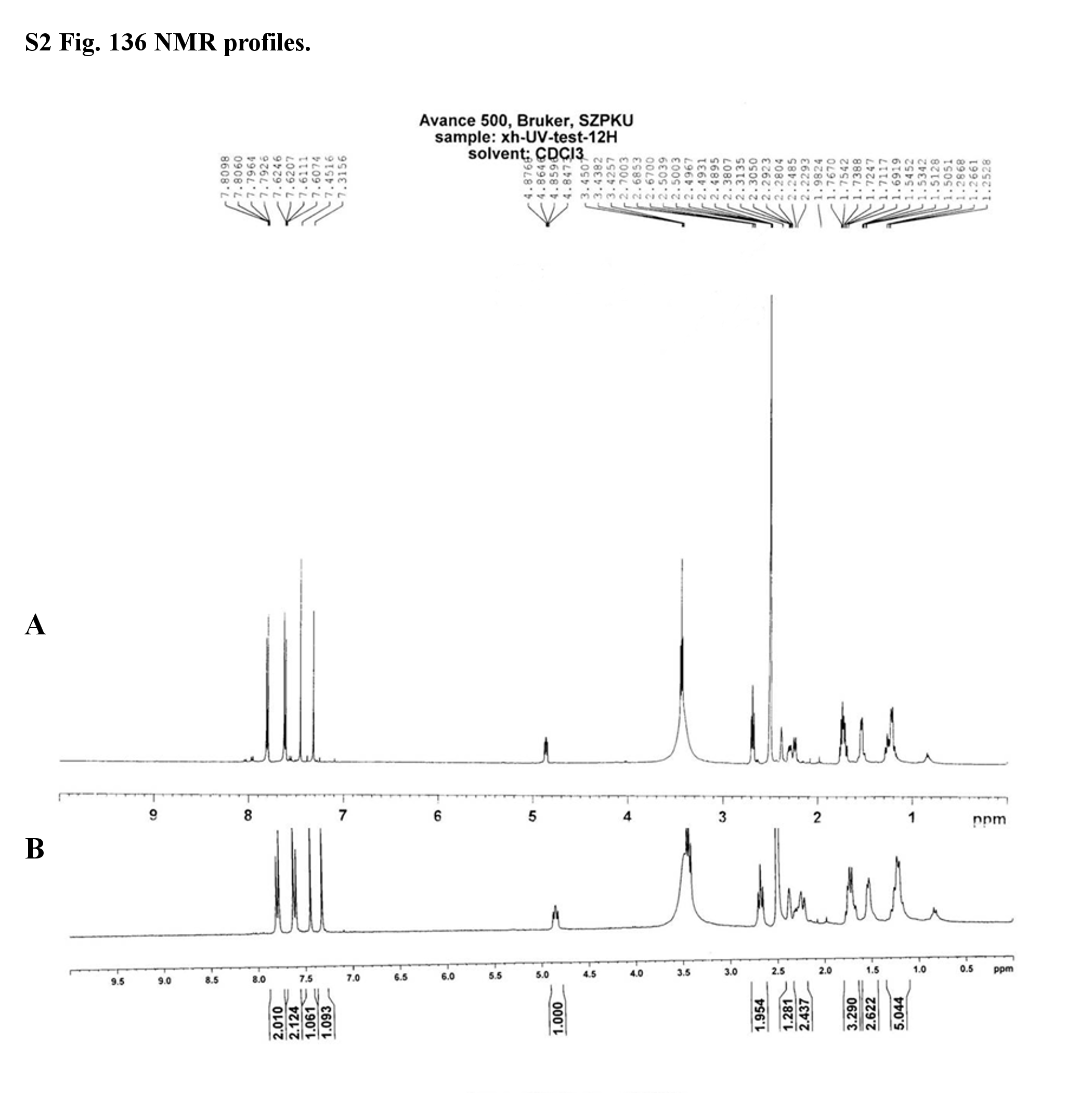

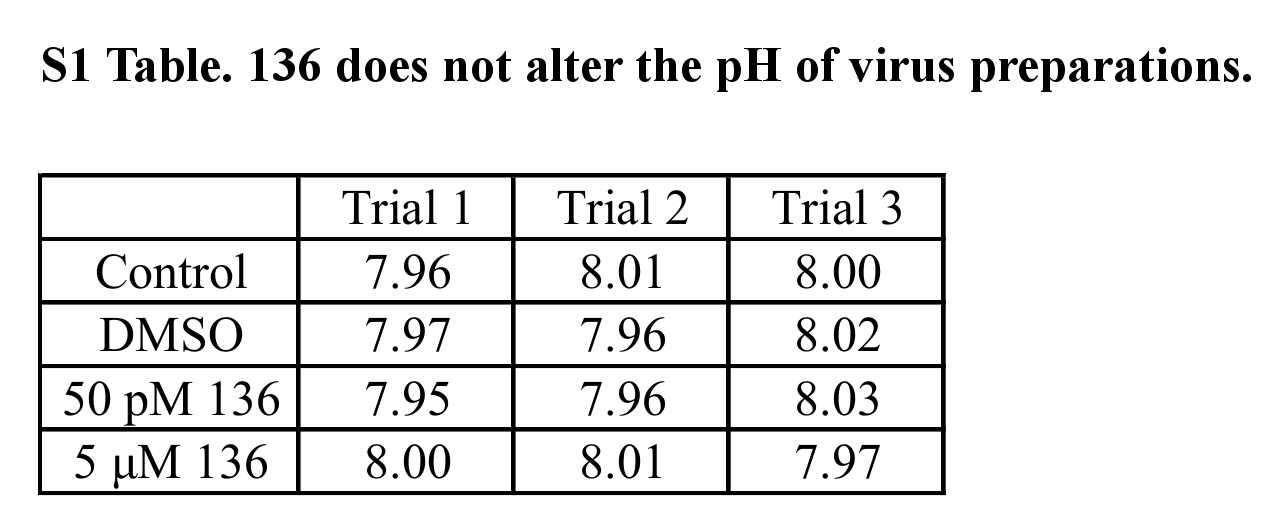

Supplement: S1 File — The detailed chemical synthesis protocols for 136 and 211. (DOCX) [file pone.0122536.s003.docx]
